# Operando Investigation of Ag‐Decorated Cu_2_O Nanocube Catalysts with Enhanced CO_2_ Electroreduction toward Liquid Products

**DOI:** 10.1002/anie.202017070

**Published:** 2021-02-22

**Authors:** Antonia Herzog, Arno Bergmann, Hyo Sang Jeon, Janis Timoshenko, Stefanie Kühl, Clara Rettenmaier, Mauricio Lopez Luna, Felix T. Haase, Beatriz Roldan Cuenya

**Affiliations:** ^1^ Department of Interface Science Fritz-Haber Institute of the Max-Planck Society Faradayweg 4–6 14195 Berlin Germany

**Keywords:** CO_2_ electroreduction, Cu_2_O, CuAg alloy, operando surface-enhanced Raman spectroscopy, operando X-ray absorption spectroscopy

## Abstract

Direct conversion of carbon dioxide into multicarbon liquid fuels by the CO_2_ electrochemical reduction reaction (CO_2_RR) can contribute to the decarbonization of the global economy. Here, well‐defined Cu_2_O nanocubes (NCs, 35 nm) uniformly covered with Ag nanoparticles (5 nm) were synthesized. When compared to bare Cu_2_O NCs, the catalyst with 5 at % Ag on Cu_2_O NCs displayed a two‐fold increase in the Faradaic efficiency for C_2+_ liquid products (30 % at −1.0 V_RHE_), including ethanol, 1‐propanol, and acetaldehyde, while formate and hydrogen were suppressed. Operando X‐ray absorption spectroscopy revealed the partial reduction of Cu_2_O during CO_2_RR, accompanied by a reaction‐driven redispersion of Ag on the CuO_x_ NCs. Data from operando surface‐enhanced Raman spectroscopy further uncovered significant variations in the CO binding to Cu, which were assigned to Ag−Cu sites formed during CO_2_RR that appear crucial for the C−C coupling and the enhanced yield of liquid products.

## Graphical abstract

Cu_2_O nanocubes showed a remarkable increase (30 %) in the Faradaic efficiency for C_2+_ liquid products after the decoration with Ag nanoparticles. Operando spectroscopy revealed a reaction‐driven redispersion of Ag on CuO_*x*_ as well as a certain Cu–Ag miscibility. These changes were coupled with significant variations in the binding of CO to Cu that favored the enhancement of the liquid product yields.

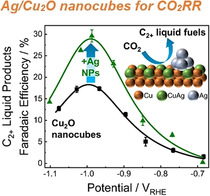

## Introduction

In the quest for developing a sustainable energy economy, the electrochemical reduction of carbon dioxide (CO_2_RR) into value‐added chemicals and fuels offers the potential to close the anthropogenic carbon cycle and store renewable (wind, solar, hydro) energy into chemical bonds.^[1]^ It has been therefore of particular interest to develop efficient and selective electrocatalysts, which reduce the reaction overpotential and steer the reaction toward hydrocarbons and multicarbon oxygenates (C_2+_). The selective generation of liquid products such as ethanol, 1‐propanol, and acetaldehyde is highly desirable due to their high energy densities and advantages for storage/transport as compared to gaseous products.^[2]^

A variety of metal electrodes can be used to catalyze CO_2_RR as demonstrated in the pioneering work by Hori.^[3]^ While some metals primarily reduce CO_2_ to CO (Ag, Au, Zn) or formate (Sn, In, Bi), copper is the only metal yielding products such as methane, ethylene, and ethanol in considerable amounts.^[4]^ However, the selective conversion to C_2+_ products in the form of liquids (alcohols and carbonyls) still requires high overpotentials, suffers from low current densities that can be achieved, and the generation of parasitic hydrogen through the competing hydrogen evolution reaction (HER). Various strategies have been developed to enhance the performance of Cu‐based catalysts, including nanostructuring Cu (control of exposed facets, defects and low‐coordinated sites),^[5]^ engineering the Cu–electrolyte interface (change of local pH),^[6]^ and adjusting the Cu oxidation state (compositional change).^[7]^ For example, Cu_2_O nanocubes (NCs) with well‐ordered (100) facets have been shown to lead to an increase in the selectivity toward ethylene, while suppressing methane production.[^5e^, ^8^]

A promising way to further improve the catalyst's performance and selectivity is the introduction of a secondary metal.^[9]^ Recent studies of Cu–Ag bimetallic systems showed enhanced selectivity for C_2+_ products. In particular, a phase‐blended Ag‐Cu_2_O catalyst had a three times higher Faradaic efficiency (FE) toward ethanol than Ag‐free Cu_2_O, but suffered from low activity (|*j*_ethanol_| <1 mA).^[10]^ Additionally, Ag‐covered Cu_2_O nanowires prepared via a galvanic replacement reaction enabled a 1.4 times higher current density toward ethylene production as compared to pure Cu_2_O nanowires.^[11]^ Furthermore, CuAg surface alloys have been found to be more selective for the formation of multi‐carbon products than pure copper.^[12]^ The facilitated yield of C_2+_ products in the bimetallic system is usually linked to the suppression of HER due to the enhanced coverage of *CO adsorbates as compared to *H,^[12b]^ and the diffusion of CO from Ag sites to Cu sites that enables C−C coupling (CO spillover).[^10^, ^11^] Note here that a short diffusion path of CO and therefore a homogeneous distribution of Cu and Ag at the surface of the catalyst are essential for an effective CO spillover.[^10^, ^12b^] Nonetheless, although the spatial arrangement of Cu and Ag in these studies seems to play a key role for the different CO_2_RR selectivity trends obtained, the key parameters for achieving an optimal synergy in Cu–Ag bimetallic systems are still unknown. In particular, open questions still remain on the composition and structure of the most active and C_2+_‐selective systems under operando CO_2_RR conditions, including the stability of Cu_2_O, which might be modified by introducing Ag.^[13]^

Herein, we prepared well‐defined Cu_2_O NCs (35 nm) uniformly covered with Ag nanoparticles (NPs, 5 nm) by a facile wet‐chemical ligand‐free synthesis. Employing ex situ, in situ and operando characterization techniques, we gained insight into the morphology, chemical state, composition, and adsorbates of the Cu–Ag catalyst under CO_2_RR conditions. In particular, we discuss reaction‐induced Ag redispersion, Cu–Ag surface alloy formation, the influence of Ag on the reduction of Cu_2_O, the adsorption of CO on Cu and Ag, and the effect of the former parameters in the CO_2_RR activity and selectivity.

## Results and Discussion

**Figure 1:**
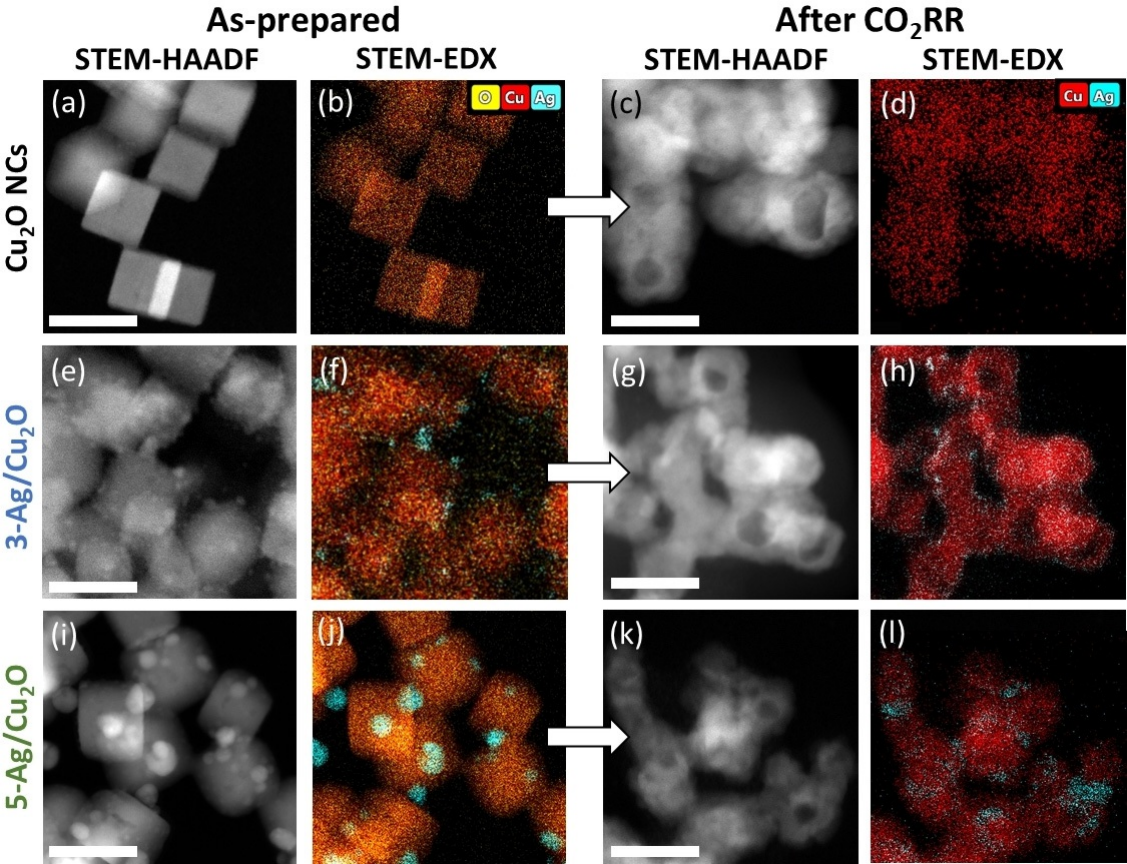
STEM‐HAADF images with corresponding EDX maps of Cu_2_O NCs, 3‐Ag/Cu_2_O, and 5‐Ag/Cu_2_O in the upper, middle, and lower panels, respectively. As‐prepared samples are shown on the left (a,e,i) with EDX maps in (b,f,j) and samples after 2 h of CO_2_RR at −1.0 V_RHE_ on the right (c,g,k) with EDX maps in (d,h,l). The scale bars correspond to 50 nm.

Figure 1 shows STEM‐HAADF and STEM‐EDX maps of pure and Ag NP‐decorated Cu_2_O NCs. The as‐prepared Cu_2_O NCs have an edge length of 35±7 nm (Figure 1 a). The STEM‐EDX data (Figure 1 b, S1, Table S1) reveal an average Cu/O at % ratio of 66:34, which corresponds to the composition of Cu_2_O. In the following, the Cu_2_O NCs decorated with 3 and 5 at % of Ag will be denoted 3‐Ag/Cu_2_O and 5‐Ag/Cu_2_O. The Ag NPs with a diameter of 4.6±1.1 nm (3‐Ag/Cu_2_O, Figure 1 e, S2) and of 6.0±2.1 nm (5‐Ag/Cu_2_O, Figure 1 i, S3) appear to be uniformly distributed on the surface of the Cu_2_O NCs. The STEM‐EDX maps of the as‐prepared 3‐ and 5‐Ag/Cu_2_O catalysts (Figure 1 f,j) indicate a clear phase separation between the Ag NPs and the Cu_2_O NCs.

After 2 h of CO_2_RR at −1.0 V_RHE_ in 0.1 m KHCO_3_, the cubic morphology appeared less pronounced and hollow structures formed in all cases (Figure 1 c,g,k). Simultaneously, the edge length of the NCs decreased in average by 3 nm (Table S2) and the size distribution broadened, as reported in the literature for pure Cu_2_O NCs after CO_2_RR.^[8]^ STEM‐EDX maps after reaction (Figure 1 d,h,l) reveal that Cu_2_O is partially reduced to metallic Cu (Table S3) and that the clear phase separation between Ag and Cu is lost. Instead, small Ag clusters are dispersed on the Cu surface. In addition, some Ag‐rich areas with sizes of 6.3±1.6 nm (3‐Ag/Cu_2_O) and 9.5±2.5 nm (5‐Ag/Cu_2_O) were also found (Table S2).

X‐ray diffraction (XRD) was applied to confirm the phase purity of the catalysts and to track the evolution of the crystal structure after CO_2_RR. Figure S4 shows the XRD pattern of the as‐prepared Cu_2_O NCs and Ag/Cu_2_O with the main Cu_2_O reflections assigned to (111) at 36.4° and (200) at 42.3°. The presence of metallic Ag can be seen by the Ag(111) at 38.2° and Ag(200) at 44.5° for the Ag/Cu_2_O. The low intensity of the *fcc* Ag reflections can be attributed to the low Ag loading and XRD peak broadening due to small particle sizes.

Table 1 shows the as‐prepared composition and coherence length derived from Rietveld refinement of the XRD patterns. The coherence length of Cu_2_O agrees well with the size distribution obtained by STEM analysis, although it is slightly smaller than the mean NC edge length. The atomic fractions of metallic Ag in the as‐prepared Ag/Cu_2_O agree well with the Cu/Ag composition obtained by ICP‐MS (Table S5). This confirms that the majority of the added Ag from the AgNO_3_ solution was incorporated in the Ag NPs, and that the initial ratios of the metal salts utilized were maintained.

**Table 1:** Atomic fraction and structural coherence length of Cu_2_O and metallic Cu and Ag phases.^[a]^

Sample	Atomic fraction [at %]			Structural coherence length [nm]		
	Cu_2_O	*fcc* Cu	*fcc* Ag	Cu_2_O	*fcc* Cu	*fcc* Ag
*As‐prepared*						
Cu_2_O NC	100	–	–	32.6(3)	–	–
3‐Ag/Cu_2_O	97.55(9)	–	2.45(6)	32.3(7)	–	5.9(7)
5‐Ag/Cu_2_O	95.1(7)	–	4.9(5)	23.1(3)	–	6(2)
						
*After CO_2_RR*						
5‐Ag/Cu_2_O	56(16)	24(9)	20(7)	6.8(3)	9(2)	4.4(5)

[a] Derived from Rietveld refinement of ex situ XRD patterns of as‐prepared Cu_2_O NCs, 3‐Ag/Cu_2_O, and 5‐Ag/Cu_2_O, and of 5‐Ag/Cu_2_O deposited on carbon paper after 2 h of CO_2_RR at −1.0 V_RHE_.

**Figure 2:**
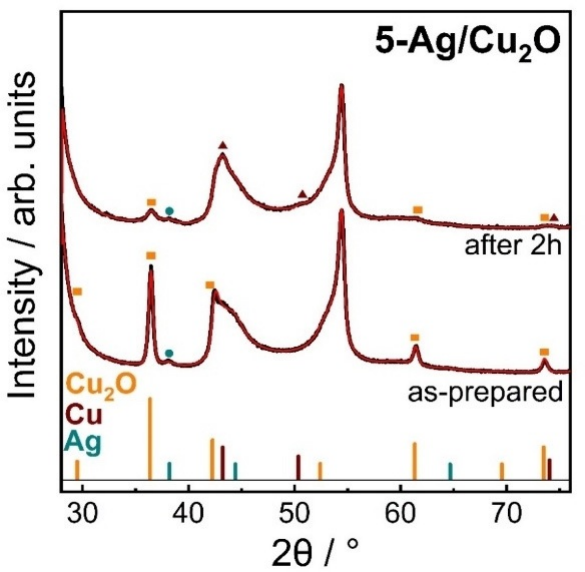
Ex situ XRD pattern of 5‐Ag/Cu_2_O deposited on carbon in the as‐prepared state and after 2 h of CO_2_RR at −1.0 V_RHE_. Red lines correspond to Rietveld fits.

The structural evolution of the 5‐Ag/Cu_2_O catalyst was investigated before and after 2 h of CO_2_RR using ex situ grazing incidence (GI) XRD (Figure 2, Table 1) and shows the reduction of Cu_2_O to metallic Cu as prominently seen in the Cu(111) reflection at 43.2°. The background arises from the carbon paper support (Figure S5). Rietveld refinement of 5‐Ag/Cu_2_O after CO_2_RR suggests a mixture of Cu_2_O and metallic Cu and a significantly increased Ag fraction, while ICP‐MS did not show changes in the catalyst composition (Table S6). Thus, we are missing a considerable fraction of Cu/CuO_*x*_ after reaction according to XRD. This suggests that Cu might be present in non‐crystalline domains, resulting in an increased Ag/Cu ratio. Additionally, there is a slight contraction of the Ag lattice (4.092±0.003 to 4.088±0.003 Å), which could be explained by the incorporation of Cu into the Ag lattice (Table S4). Notably, the coherence length of Cu_2_O decreased strongly from 23.1 nm (as‐prepared) to 6.8 nm (after CO_2_RR), reaching a similar value to the coherence length of the metallic Cu phase (9 nm). We conclude that the hollow structures after CO_2_RR (Figure 1) might consist of a mixture of Cu_2_O and Cu crystallites as seen in XRD. The coherence length of the metallic Ag phase is on average slightly decreased after CO_2_RR, which agrees with the partial re‐dispersion of Ag on the Cu surface and the Ag‐rich domains likely being formed from multiple Ag NPs.

**Figure 3:**
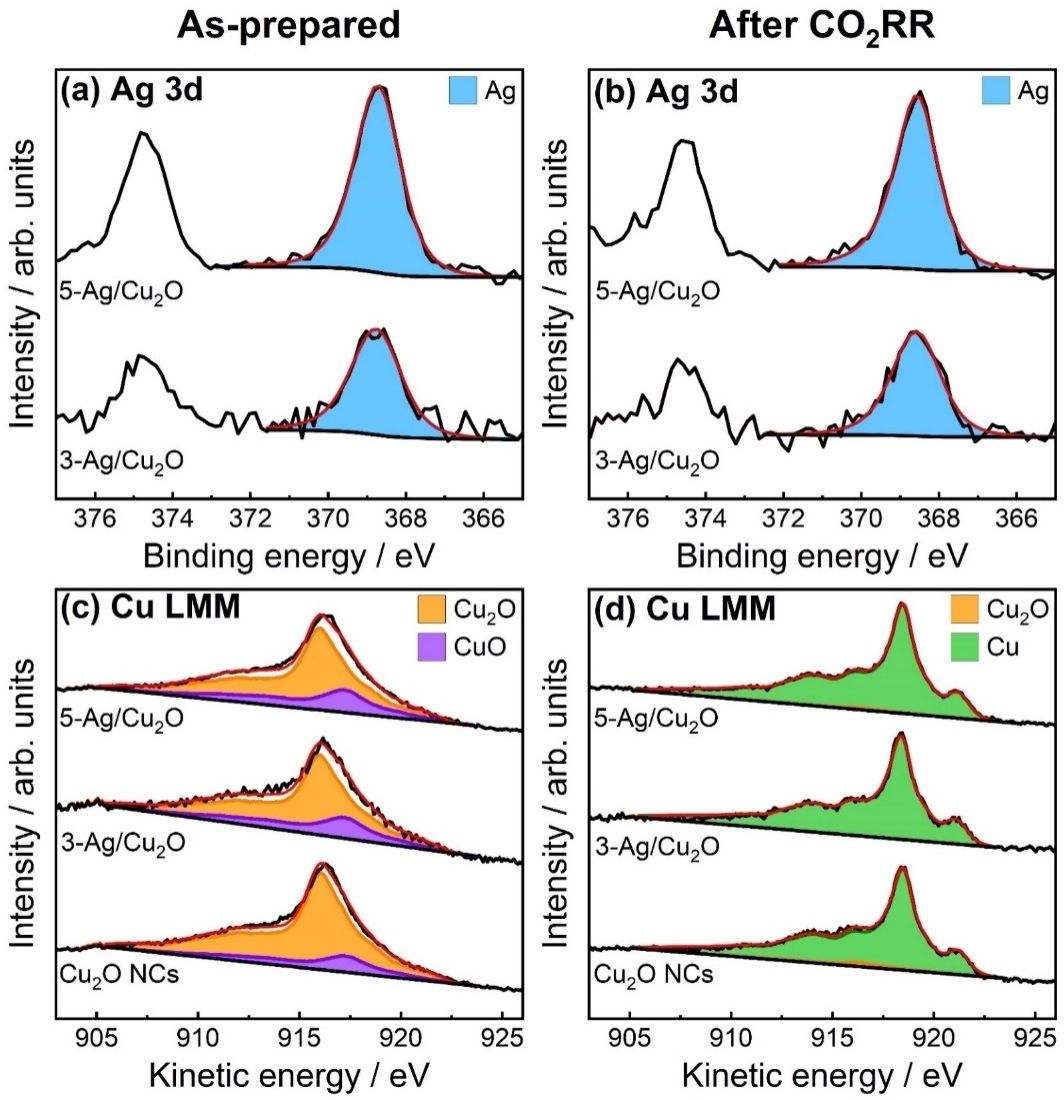
Quasi‐in situ Ag 3d XPS and Cu LMM XAES spectra of Cu_2_O NCs, 3‐Ag/Cu_2_O, and 5‐Ag/Cu_2_O (a,c) in the as‐prepared state and (b,d) after 2 h of CO_2_RR at −1.0 V_RHE_ (without air exposure) with the corresponding fits (red line) and reference spectra.

Quasi‐in situ X‐ray photoelectron spectroscopy (XPS) measurements were performed to gain deeper insight into the surface composition and chemical state of the Ag NP‐decorated and pure Cu_2_O NCs before and after 2 h of CO_2_RR. Figure 3 presents the Ag 3d and Cu Auger LMM spectra, while Figure S6 shows the Cu 2p spectra. The Ag 3d core level regions of 3‐ and 5‐Ag/Cu_2_O (Figure 3 a,b) reveal that Ag is in the metallic state before and after CO_2_RR, which is also consistent with the XRD results. The Ag/Cu surface composition ratios were determined by integrating the peak areas of Ag 3d_5/2_ and Cu 2p_3/2_. As expected for Ag NPs decorating the surface of Cu_2_O NCs, the Ag/Cu ratio extracted from the more surface‐sensitive XPS technique was higher than the bulk composition obtained from the XRD and ICP‐MS analysis. After CO_2_RR, the Ag/Cu XPS ratios further increased from 4:96 to 7:93 at % in 3‐Ag/Cu_2_O and from 9:91 to 11:89 in 5‐Ag/Cu_2_O. Thus, we conclude that a more homogeneous distribution of Ag (redispersion) on the Cu_2_O surface takes place during CO_2_RR. Furthermore, we can exclude significant preferential dissolution of Cu during CO_2_RR due to the constant bulk Ag/Cu ratio before and after CO_2_RR as demonstrated by ICP‐MS (Table S6). The higher Ag signal observed after CO_2_RR agrees with the formation of smaller Ag NPs and clusters and an enhanced Ag dispersion, as also revealed by the STEM and XRD data.

Additionally, deconvolution of the Cu LMM spectra (Figure 3 c,d, Table S7) was carried out to distinguish Cu^0^, Cu^I^, and Cu^II^ near‐surface species. In the as‐prepared state, the samples consisted mainly of Cu_2_O with a contribution of CuO. After 2 h of CO_2_RR, the near‐surface regions of the Cu_2_O NCs and Ag/Cu_2_O samples were fully reduced to metallic Cu within the error margin. In contrast to prior studies, our samples were not exposed to air after CO_2_RR, since our electrochemical cell is directly connected to the XPS chamber. Thus, even though no potential is applied during the XPS measurement, re‐oxidation in air can be excluded.

**Figure 4:**
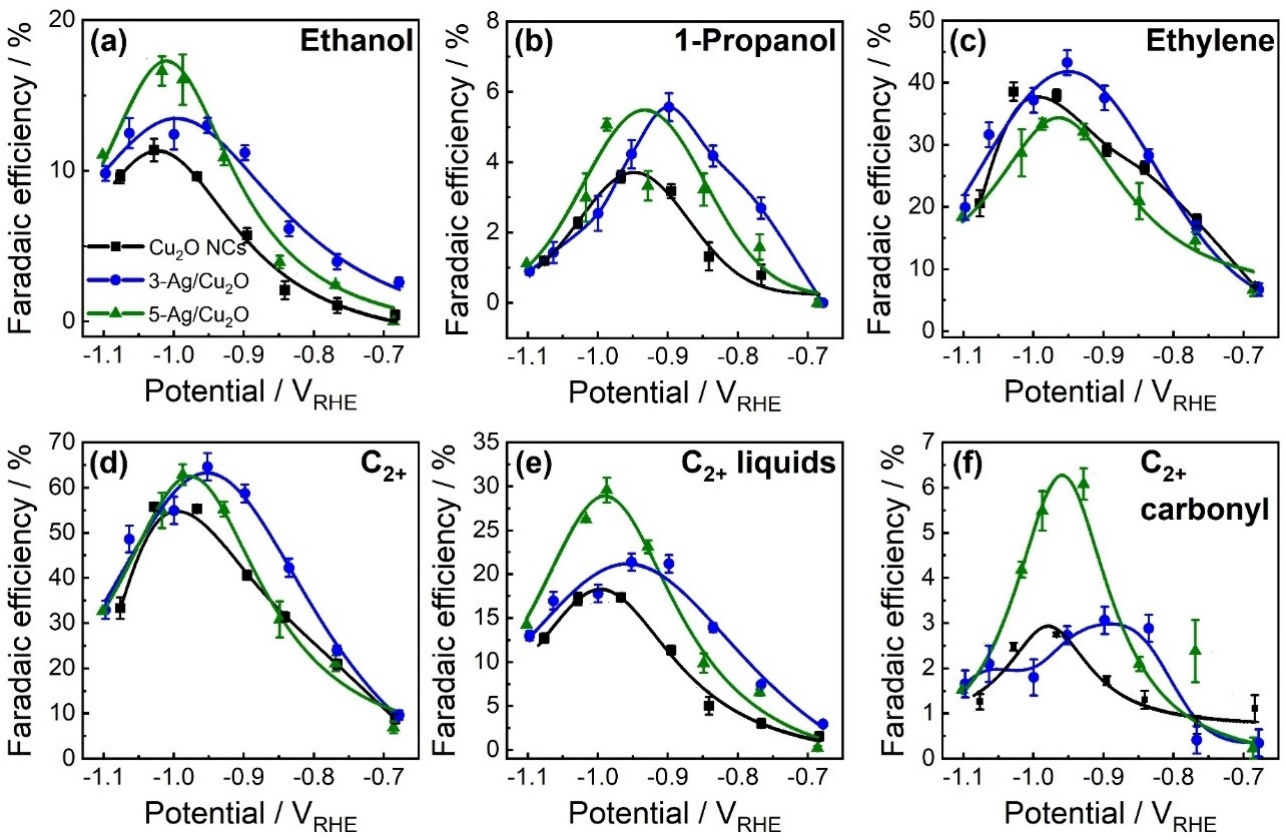
Potential‐dependent Faradaic efficiencies of a) ethanol, b) 1‐propanol, c) ethylene, d) C_2_+C_3_ products (C_2+_), e) C_2+_ liquid products, and f) C_2+_ carbonyl products of Cu_2_O NCs (black), 3‐Ag/Cu_2_O (blue), and 5‐Ag/Cu_2_O (green) after 2 h of electrolysis in CO_2_‐saturated 0.1 m KHCO_3_. Solid lines are a guide for the eye.

The electrocatalytic performance of Ag NP‐decorated and pure Cu_2_O NCs deposited on carbon paper was evaluated by chronoamperometric measurements for 2 h at potentials between −0.7 and −1.1 V_RHE_ in a CO_2_‐saturated 0.1 m KHCO_3_ electrolyte (Figures 4, S7–S11, Table S8). Figure 4 displays the Faradaic efficiencies (FEs) of the main C_2_ and C_3_ products, namely, ethanol, 1‐propanol, and ethylene, as well as the combined FEs of all C_2+_ products, of the C_2+_ liquid products, and of the C_2+_ carbonyl products; see also Figures S8 (C_1_ products), S9 (minor C_2+_ products), S10 (sum of all liquid products), and S11 (geometric and mass current densities).

The production of ethanol at −1.0 V_RHE_ increases significantly with the amount of Ag. Cu_2_O NCs, 3‐, and 5‐Ag/Cu_2_O reach 10 %, 13 %, and 17 % FE for ethanol at −1.0 V_RHE_, respectively (Figures 4 a, S7a). Thus, FE_ethanol_ increased 1.5 times by the addition of 5 at % of Ag to Cu_2_O NCs. Additionally, the production of 1‐propanol is doubled in the Ag/Cu_2_O samples with FE of 5–6 % at −0.9 V_RHE_ compared to 3 % for the Cu_2_O NCs (Figure 4 b). This increase in alcohol yield has been previously linked to CO spillover from Ag to Cu, since the weaker binding between Ag and the *CO intermediate is considered to facilitate CO production as compared to the moderate binding energy between Cu and the *CO intermediate leading to the formation of hydrocarbons.[^4^, ^10^] Also in our case, the Ag/Cu_2_O samples show higher FEs for CO at low overpotentials, with almost 40 % FE at −0.7 V_RHE_ in comparison to 20 % FE for the pure Cu_2_O NCs (Figure S8b). At higher overpotentials, the FE_CO_ decreases for all catalysts, and starting from −1.0 V_RHE_ the selectivity for CO of the Ag/Cu_2_O is even similar to that of the bare Cu_2_O NCs (FE_CO_=3–5 %). We note that 3‐Ag/Cu_2_O shows a better ability to transform CO_2_ into C_2+_ products at lower overpotentials than 5‐Ag/Cu_2_O, which is also related to a lower FE of CO starting from −0.83 V_RHE_ (Figure S8b). These differences might originate from the different sizes of the Ag NPs in the two samples as extracted by STEM analysis (Table S2), which can affect the electrocatalytic reduction of CO_2_ to CO as well as the CO spillover to the Ag/Cu interface.

The production of ethylene on the Cu_2_O NCs peaks at −0.95 V_RHE_ with 40 % FE (Figures 4 c, S7c), while for the 3‐Ag/Cu_2_O the FE is slightly larger (45 %). In contrast, 5‐Ag/Cu_2_O shows a lower FE_ethylene_ (34 %), paralleled by an enhancement in the ethanol production. Consequently, the FE_ethanol_/FE_ethylene_ ratio at −1.0 V_RHE_ increases with Ag loading from 0.28 for Cu_2_O NCs, 0.33 for 3‐Ag/Cu_2_O, to 0.49 for 5‐Ag/Cu_2_O. The C_2+_ selectivity is the highest for the two Ag/Cu_2_O samples with a FE of 65 % at −0.98 V_RHE_, which is an increase of 10 % compared to pure Cu_2_O NCs (Figure 4 d, S7d).

Furthermore, the parasitic HER (Figure S8a) and the production of formate (the only C_1_ liquid, Figure S8d) were (slightly) suppressed. The latter has also been recently observed for AgCu foam catalysts as compared to pure Cu foams.^[14]^ In contrast, the formation of acetaldehyde (Figure S9a) and propionaldehyde (Figure S9b) is significantly increased for 5‐Ag/Cu_2_O, which results in an enhancement of the carbonyl C_2+_ products by a factor of three (Figure 4 f, S7f). Overall, introducing Ag increases the FE of the desired C_2+_ liquid products (Figure 4 e, S7e) for 5‐Ag/Cu_2_O by 15 % in comparison to Cu_2_O NCs at −1.0 V_RHE_. This is assigned to the enhanced production of ethanol, 1‐propanol, allyl alcohol, and carbonyl C_2+_ products (Figure 4 a,b,f, S9c). We additionally performed long‐term CO_2_RR measurements for 12 h at −1.0 V_RHE_ to track the stability of the catalytic performance (Figure S12) and found a good stability for all catalysts. For the Ag/Cu_2_O samples, the amount of C_2+_ liquid products remains stable over time with a decrease of the aldehydes and increase of 1‐propanol as well as acetate for the 5‐Ag/Cu_2_O catalyst.

In order to further examine the Cu–Ag interaction and its role in the CO_2_RR selectivity, we also investigated the catalytic performance of the pure Cu_2_O NCs deposited on a polished Ag foil (Cu_2_O/Ag). In this configuration (Figure S13), the production of C_2+_ liquids is also enhanced on the Cu_2_O/Ag sample at −1.0 V_RHE_, indicating again the importance of the CO generated at the Ag sites for the further hydrogenation of the C−C products generated on the Cu sites. However, the Cu_2_O/Ag sample shows a drastic increase of HER as compared to the 5‐Ag/Cu_2_O, namely from 20 to 40 % FE, and a suppression of the ethylene FE from 30 % to 20 %. This might be correlated to the decrease of the total area of the Ag/Cu interface, which decreases the atomic interaction and synergy between Ag and Cu. Consequently, the well‐distributed Ag NPs, which redispersed on the surface of our Cu_2_O NCs, appear to play a significant role in the observed synergistic effect and the C−C coupling mechanism.

**Figure 5:**
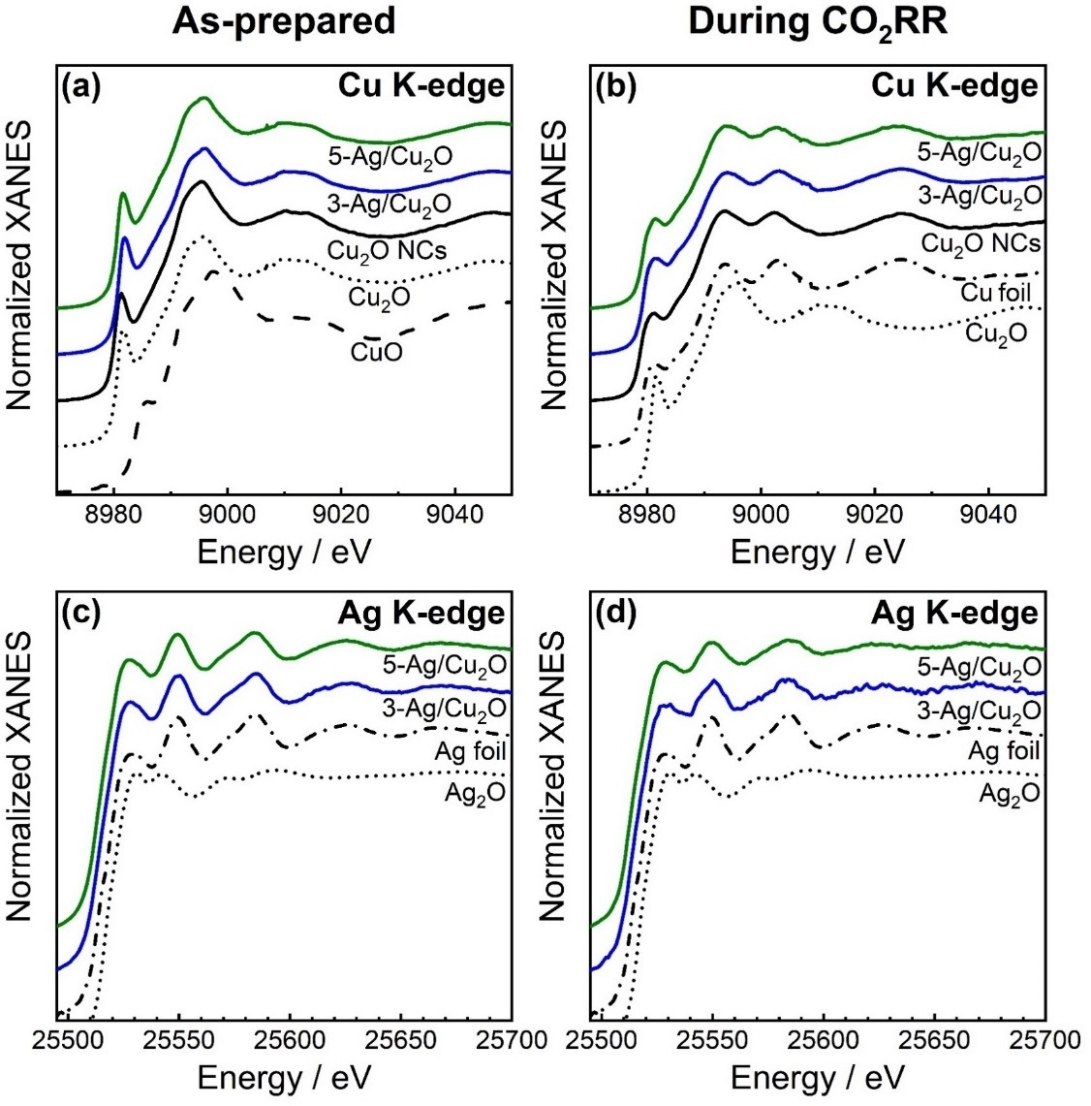
Normalized Cu and Ag K‐edge XANES spectra of Cu_2_O NCs (black), 3‐Ag/Cu_2_O (blue), and 5‐Ag/Cu_2_O (green) in as‐prepared state (a,c) and in the final state under CO_2_RR at −1.0 V_RHE_ (b,d). Reference spectra of Cu_2_O, CuO, Cu foil, Ag_2_O, and Ag foil are shown for comparison.

Further insight into the Ag–Cu interaction can be extracted from operando X‐ray absorption spectroscopy (XAS) data. Figure 5 depicts the normalized Cu K‐edge and Ag K‐edge X‐ray absorption near edge structure (XANES) spectra of the Ag NP‐decorated and pure Cu_2_O NCs in their as‐prepared state (Figure 5 a,c) and during CO_2_RR in a steady‐state after 5 h of the reaction (Figure 5 b,d). The position of the absorption edge in the Cu K‐edge XANES spectra of the as‐prepared Cu_2_O NCs compared to the reference spectra shows that the NCs mainly exhibit a Cu^I^ oxidation state with the characteristic pre‐edge feature at 8981 eV. Linear combination analysis (LCA) of Cu K‐edge XANES data revealed the presence of Cu^I^ and Cu^II^ species (Figure S14), as already seen in the more surface‐sensitive XPS analysis. The decoration with the Ag NPs does not change the Cu XANES spectra (Figure 5 c). Ag K‐edge XANES spectra, in turn, confirm the metallic state of Ag with all XANES features resembling those of the Ag foil. These findings are consistent with the STEM, XRD, and XPS results, emphasizing the lack of significant interaction between the Cu_2_O and Ag species in the as‐prepared state.

The reduction of the Cu_2_O NCs was investigated during CO_2_RR at −1.0 V_RHE_ for 5 h (Figure S14). In the final state, the Cu K‐edge XANES spectrum resembles that of the metallic Cu foil reference (Figure 5 b). We tracked the evolution of the Cu^0^/Cu^I^/Cu^II^ ratios by LCA (Figure S14b,c) and our analysis revealed that a significant fraction of Cu^I^ was preserved under CO_2_RR reaction conditions with 15–25 % of Cu^I^ present in all samples. Figure 5 d shows the Ag K‐edge XANES spectra under CO_2_RR conditions at −1.0 V_RHE_ after reaching stationary conditions. Ag remained metallic, but an attenuation of the post‐edge oscillations is visible during CO_2_RR, explainable by a decrease in the size of the Ag NPs due to their redispersion on the Cu_2_O NC surface.^[15]^

**Figure 6:**
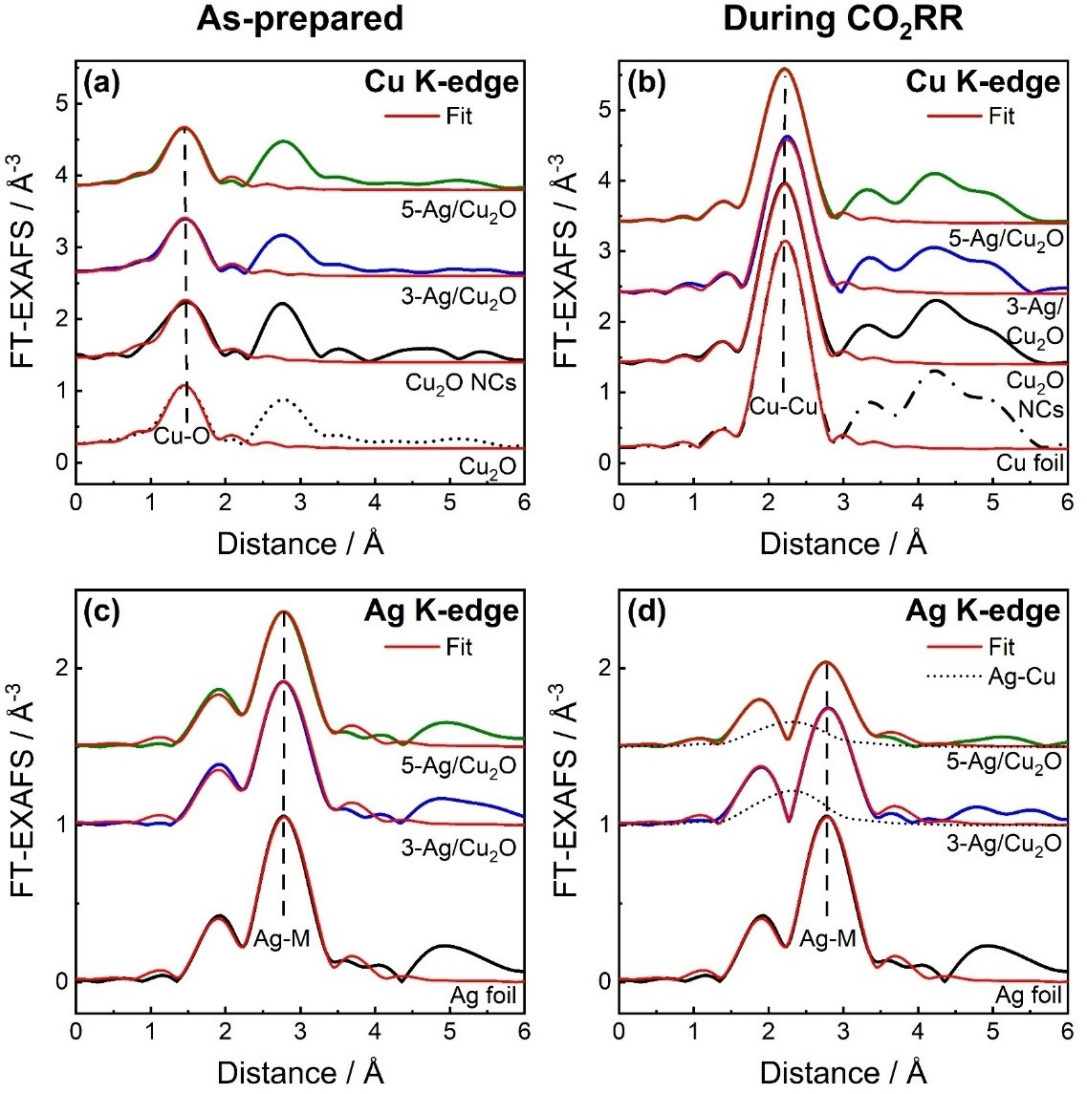
Moduli of Fourier‐transformed Cu and Ag K‐edge EXAFS spectra of Cu_2_O NCs (black), 3‐Ag/Cu_2_O (blue), and 5‐Ag/Cu_2_O (green) in the as‐prepared state (a,c) and in the final state under operando CO_2_ reduction conditions at −1.0 V_RHE_ (b,d) with corresponding fits (red). Partial contribution of Ag−Cu bonds, as obtained from EXAFS data fitting, is also shown in (d) with dotted lines. Reference spectra of Cu_2_O, Cu and Ag foils are shown for comparison.

To achieve a deeper understanding of the local atomic structure, Fourier transformed extended X‐ray absorption fine structure (FT‐EXAFS) spectra of the Ag NP‐decorated and pure Cu_2_O NCs are shown in Figure 6 with the corresponding Fourier‐filtered EXAFS spectra in Figure S15. The peaks at 1.5 Å and 2.8 Å (phase uncorrected) in Cu K‐edge FT‐EXAFS and at 2.8 Å in Ag K‐edge FT‐EXAFS for the as‐prepared samples indicate the presence of Cu−O and Cu−Cu bonds (typical for Cu_2_O) and Ag−M bonds (here, M is Ag or Cu), respectively, in agreement with the XANES analysis.

Under CO_2_RR conditions at −1.0 V_RHE_, a strong peak at 2.2 Å (phase‐uncorrected) appeared in the Cu K‐edge FT‐EXAFS, which can be attributed to the Cu−Cu contribution in metallic Cu. The Ag−M peak position at 2.8 Å in Ag K‐edge FT‐EXAFS did not change significantly, and no additional peaks appeared. Nonetheless, the comparison of the FT‐EXAFS before and during CO_2_RR shows a decrease in the Ag−M peak intensities. Moreover, the intensity of both, the Cu−Cu and Ag−M peaks decreases with an increase in Ag loading (Figure 6). These differences suggest structural changes in the Ag and Cu atomic environment under CO_2_RR conditions, which we further investigated using quantitative fitting of the EXAFS spectra to obtain the structural parameters presented in Tables S10–S11. For the as‐prepared state, we obtained a Cu−O coordination number (*N*_Cu−O_) of circa 2 and a Cu−O distance (*R*_Cu−O_) of circa 1.87 Å, which agree with the values of these parameters in bulk Cu_2_O. Furthermore, in the as‐prepared samples, the *N*_Ag−Ag_ decreased from 12 in the Ag foil over 11.4±0.4 in 3‐Ag/Cu_2_O to 10.5±0.3 in 5‐Ag/Cu_2_O, indicating an enhanced disorder within the Ag NPs. However, the *R*_Ag−Ag_ distances in all samples are comparable to that of the Ag foil, 2.833±0.003 Å, which means that there is no significant lattice contraction due to alloying in the as‐prepared state.

Under stationary CO_2_RR conditions, the Cu−Cu coordination number of approx. 9 is lower than that of the Cu foil reference (12). We also observe Cu−O bonds with *N*_Cu−O_≈0.3. The Cu−Cu distance (*R*_Cu−Cu_) during CO_2_RR, 2.524±0.003 Å, is comparable to that in the bulk Cu foil reference (2.527±0.002 Å). The Cu^0^/Cu^I^ ratio under CO_2_RR, as obtained from the EXAFS analysis, agrees well with the XANES data (Table S9), showing the partial reduction of the Cu_2_O to metallic Cu under CO_2_RR conditions. Our primary finding from the Cu K‐edge is the increasing disorder of the Cu−Cu bonds with higher Ag loading and thus reveals the decoration of the Cu_2_O NCs with Ag NPs during CO_2_RR, but one cannot exclude that this could also be a result of incomplete CuO_*x*_ reduction.

One of the main goals of this study is to explore the interaction between Cu and Ag at the atomic scale under CO_2_RR conditions. Due to the low concentration of Ag as compared to Cu, information on the interplay between Ag and Cu atoms can be best extracted from the analysis of Ag EXAFS data (Figure 6 c,d, S16). During CO_2_RR, we identified an additional contribution of Ag−Cu bonds with *R*_Ag−Cu_ of 2.623±0.005 Å (3‐Ag/Cu_2_O) and 2.596±0.008 Å (5‐Ag/Cu_2_O), while the bond lengths in the more prominent Ag−Ag component did not contract significantly for 3‐Ag/Cu_2_O (2.840±0.005 Å), but did so for 5‐Ag/Cu_2_O (2.787±0.007 Å). The Ag−Ag coordination number decreased from 12 (bulk) to 9, and an Ag−Cu coordination number (*N*_Ag−Cu_) of up to 1 was obtained under CO_2_RR conditions, being larger for the 3‐Ag/Cu_2_O sample than for 5‐Ag/Cu_2_O. The obtained Ag−Cu bond length is in between the values for the Cu−Cu distance in bulk Cu (2.527±0.002 Å) and the Ag−Ag distance in bulk Ag (2.833±0.003 Å). The Ag−Cu bond lengths as well as the coordination numbers suggest the partial incorporation of Ag into Cu‐rich domains either as an AgCu phase or as dispersed particles or clusters on the Cu surface under CO_2_RR conditions. Furthermore, the reduced total Ag−M coordination numbers with respect to those in the as‐prepared samples suggest an increase in the disorder in the Ag−Ag component during CO_2_RR, which agrees well with a smaller particle size and/or Ag redispersion during CO_2_RR, as shown by the STEM results. Thus, we observe the partial miscibility of Cu and Ag under CO_2_RR conditions by using operando Ag K‐edge XAS, which was not provided in comparable studies so far.

**Figure 7:**
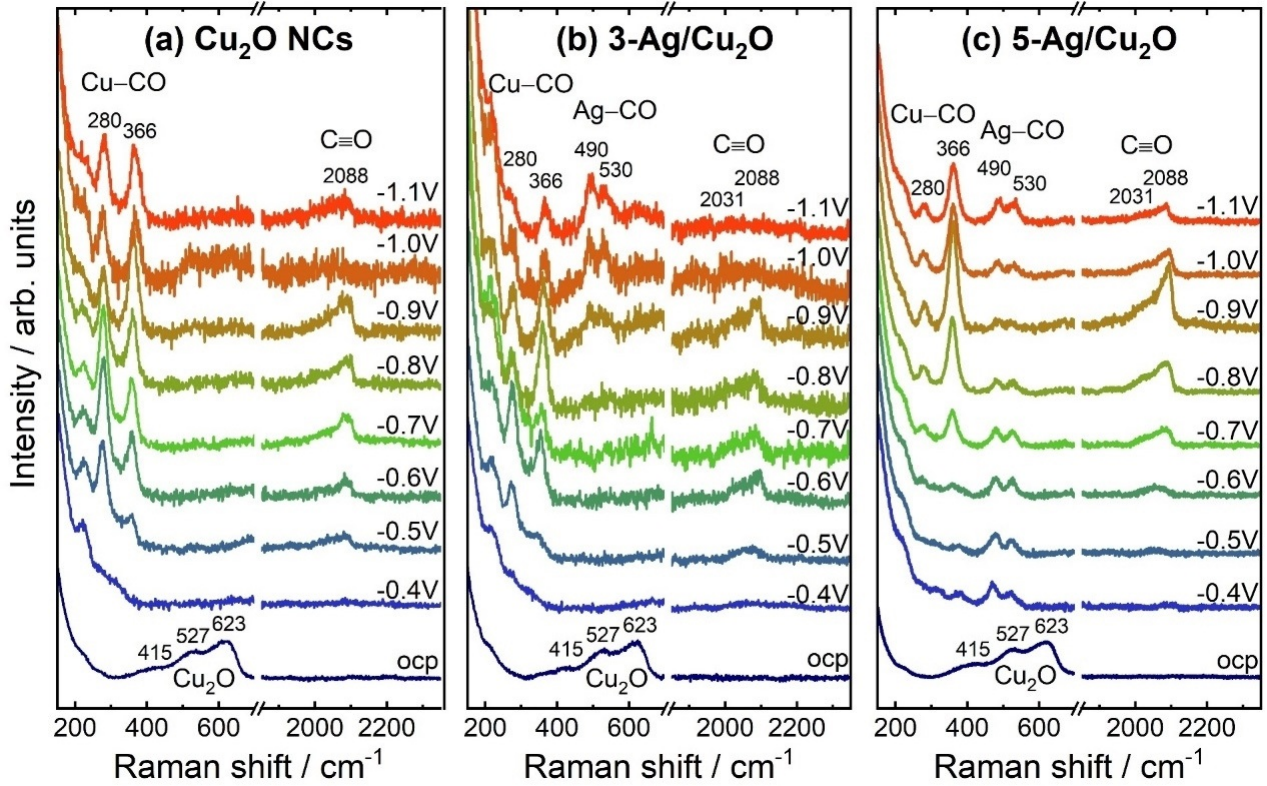
Operando surface‐enhanced Raman spectra of a) Cu_2_O NCs, b) 3‐Ag/Cu_2_O, and c) 5‐Ag/Cu_2_O at open circuit potential (ocp) and under different applied potentials in CO_2_‐saturated 0.1 m KHCO_3_. All potentials are given with reference to RHE.

We furthermore followed the reduction of the Cu_2_O species and the CO or CO‐like intermediates chemisorbed on Cu and Ag at different potentials and during CO_2_RR via operando surface‐enhanced Raman spectroscopy (SERS). Figure 7 presents potential‐dependent SERS spectra of the Ag NP‐decorated and pure Cu_2_O NCs acquired at open circuit potential (ocp) and between −0.4 and −1.1 V_RHE_ from 150–700 cm^−1^ and 1850–2350 cm^−1^ (see also Figure S17).

In the as‐prepared state, the characteristic peaks of Cu_2_O are observed at 415 cm^−1^, 527 cm^−1^, and 623 cm^−1^.^[16]^ The Cu_2_O peaks vanished for all samples after applying a reductive potential of −0.4 V_RHE_, which indicates a prompt reduction of the surface Cu_2_O species independently from the Ag loading. The latter is in agreement with the quasi‐in situ XPS analysis.

At more negative potentials, the operando SERS data of the Ag NP‐decorated Cu_2_O NCs exhibit significant differences compared to the bare Cu_2_O NCs during CO_2_RR: 1) At higher Raman shifts, a broad band of the C−O vibrations appeared at 2088 cm^−1^ for Cu_2_O NCs during CO_2_RR, while for Ag/Cu_2_O a shoulder at 2031 cm^−1^ is more pronounced. This might be assigned to the multisite binding mechanism on the AgCu surface, where each binding configuration has a different electron back‐donating ability.^[17]^ 2) For the Ag NP‐decorated Cu_2_O NC samples, two additional peaks at 490 and 530 cm^−1^ appeared, which might be assigned to Ag−CO vibrations.[^11^, ^18^] 3) Two peaks evolve differently at 280 cm^−1^ and 366 cm^−1^, caused by the Cu−CO frustrated rotation and stretching vibration, respectively.[^16b^, ^18^] Thus, we can track the CO adsorbed on Cu and Ag separately by using operando SERS and identified significant differences in the CO adsorption characteristics on Cu in the presence of dispersed Ag atoms during CO_2_RR as, interestingly, the peak intensity ratio of the two Cu−CO Raman peaks shifts towards the Cu−CO stretching vibration with decreasing electrode potential. A similar trend can also be seen with increasing Ag content at −1.0 V_RHE_. These drastic changes might originate from the way that CO predominantly binds to Cu. While on pure Cu_2_O NCs the CO binding configuration is similarly prone to Cu−CO rotation and stretching, the presence of Ag sites gives rise to a CO binding configuration that facilitates the Cu−CO stretching with thus stronger lateral confinement. We anticipate that the latter plays a critical catalytic role in enhancing the C−C coupling of neighboring CO adsorbates and thus increases the C_2+_ liquid product formation.

From the ex situ and operando studies performed under CO_2_RR we gained a detailed insight into the structural evolution of the Ag NP‐decorated Cu_2_O NC catalysts. In their as‐prepared state, the Cu_2_O NCs as well as the Ag NP‐decorated samples mainly consist of Cu_2_O, with some contribution from CuO. During CO_2_RR, Cu_2_O is partially reduced to metallic Cu, while the contribution of CuO vanishes. In fact, the operando XANES data evidenced the incomplete reduction of Cu_2_O in all samples after five hours of CO_2_RR (up to 30 % of Cu_2_O remained in the 3‐Ag/Cu_2_O sample). However, we did not find a correlation between the content of Cu_2_O (in the bulk, XAS data) and the increased selectivity for C_2+_ liquid products, which might be due to the complete reduction (XPS, SERS) of the surface Cu_2_O species to metallic Cu during CO_2_RR.

Since the fraction of Ag in the near‐surface is low (<11 at %), intermixing is plausible although a precise determination of the Ag/Cu ratio in the Ag_*x*_Cu_1−*x*_ regions is not possible. Therefore, our system consists of three classes of potentially active sites during CO_2_RR: Ag/AgCu/Cu.

To further understand how the structural/chemical rearrangements influence the reaction mechanism, it is useful to correlate such changes with the formation and stability of different intermediates during CO_2_RR. The production of CO increased at lower overpotentials with increasing Ag content in the samples.^[19]^ It has been suggested that *CO, which is a key intermediate for the C−C coupling mechanism,^[19]^ might couple with *CH_*x*_ ^[20]^ to form the *CH_2_CHO intermediate, which plays a central role in the formation of C_2+_ liquid products.^[21]^ The *CH_2_CHO intermediate can be hydrogenated to *CH_3_CHO and form acetaldehyde [Eq. 1] or be further hydrogenated to yield ethanol [Eq. 2].

(1) $${}^{*}\mathrm{CH}{}_{2}\mathrm{CHO}+H{}^{+}+e{}^{-}\to {}^{*}\mathrm{CH}{}_{3}\mathrm{CHO}\to \mathrm{CH}{}_{3}\mathrm{CHO}$$

(2) $${}^{*}\mathrm{CH}{}_{3}\mathrm{CHO}+2\;H{}^{+}+2\;e{}^{-}\to C{}_{2}H{}_{5}\mathrm{OH}$$

If *CH_3_CHO couples with another *CO, propionaldehyde [Eq. 3] and 1‐propanol [Eq. 4] can be formed.

(3) $${}^{*}\mathrm{CH}{}_{3}\mathrm{CHO}+\mathrm{CO}+2\;H{}^{+}+2\;e{}^{-}+H{}_{2}O\to {}^{*}\mathrm{CH}{}_{3}\mathrm{CH}{}_{2}\mathrm{CHO}+H{}_{2}O\to \mathrm{CH}{}_{3}\mathrm{CH}{}_{2}\mathrm{CHO}$$

(4) $${}^{*}\mathrm{CH}{}_{3}\mathrm{CH}{}_{2}\mathrm{CHO}+2\;H{}^{+}+2\;e{}^{-}\to 1\text{-}C{}_{3}H{}_{7}\mathrm{OH}$$

On Cu surfaces, acetaldehyde and propionaldehyde are usually hydrogenated to ethanol, and 1‐propanol is only detected with FE≈1 %, as seen for the Cu_2_O NCs. Therefore, we can relate the enhancement of the two aldehyde selectivities to the dispersion of the Ag atoms/small clusters on the Cu surface during CO_2_RR, which induces locally strained Cu sites with expanded Cu−Ag distances compared to Cu−Cu and, most importantly, differences in the predominant CO binding motifs. DFT calculations of our previous study showed that an expanded Cu lattice increases the binding energies for the intermediates of CO_2_RR (e.g., *CO on Cu).^[9c]^ Additionally, Ag incorporation in Cu weakens the binding energies of the reduced aldehyde intermediates and inhibits their further reduction to ethanol and 1‐propanol as demonstrated in a recent DFT study.^[22]^ However, the structural analysis of the catalysts showed that the surface partially consists of Cu/Ag areas, which also leads to the formation of ethanol and 1‐propanol at the Cu sites, since they are expected to have higher binding energies for the oxygenated intermediates as compared to the Ag–Cu mixed regions. Therefore, having Ag/AgCu/Cu interfaces as active surface sites appears to enhance the yield of C_2+_ liquid products.

## Conclusion

In summary, Ag NP‐decorated Cu_2_O nanocubes displayed enhanced selectivity toward C_2+_ liquid products, while the production of formate and hydrogen was suppressed. By means of ex situ, quasi*‐*in situ, and operando spectroscopy studies under CO_2_RR conditions, we could gain insight into the structural and chemical transformations of these catalysts that were shown to influence the selectivity trends. In particular, we found the redispersion of the Ag NPs on Cu and a certain Cu–Ag miscibility, leading to expanded Cu–Ag distances compared to metallic Cu–Cu distances. Such structural rearrangements were found to result in an enhanced formation of alcohols and aldehydes.

By comparing the selectivity of pure Cu_2_O and Ag NP‐decorated Cu_2_O NCs we concluded that even though Cu_2_O species were partially preserved under reaction conditions, they are not the sole species responsible for the enhancement in the C_2+_ liquid products, which is favored when large Ag/Cu interfaces are formed. Importantly, we correlate the enhancement to variations in the predominant CO adsorption motif on Cu in the presence of dispersed Ag atoms.

Our work contributes to the fundamental understanding of the CO_2_RR by highlighting the intricate interplay of different parameters affecting the selectivity. These include the content of residual Cu_2_O species, the presence of a secondary metal near Cu where efficient CO spillover can take place, and the alloying of Cu with another metal that is able to locally increase the interatomic distance, leading to a change in the binding energies of adsorbates and intermediates, thus favoring the formation of C_2+_ liquid products.

## Conflict of interest

The authors declare no conflict of interest.

## Supporting information

As a service to our authors and readers, this journal provides supporting information supplied by the authors. Such materials are peer reviewed and may be re‐organized for online delivery, but are not copy‐edited or typeset. Technical support issues arising from supporting information (other than missing files) should be addressed to the authors.

Supplementary
