# Supplementary material for: Operando Investigation of Ag‐Decorated Cu2O Nanocube Catalysts with Enhanced CO2 Electroreduction toward Liquid Products
Source: Angew Chem Int Ed Engl. 2021 Feb 22;60(13):7426–35. doi: 10.1002/anie.202017070 (PMC8048979; doi:10.1002/anie.202017070)
Supplement: Supplementary file 1 — Supplementary [file ANIE-60-7426-s001.pdf]

Angewandte Chemie
Eine Zeitschrift der Gesellschaft Deutscher Chemiker

Supporting Information

Operando Investigation of Ag-Decorated Cu_2O Nanocube Catalysts with Enhanced CO_2 Electroreduction toward Liquid Products

*Antonia Herzog, Arno Bergmann, Hyo Sang Jeon, Janis Timoshenko, Stefanie Kühl, Clara Rettenmaier, Mauricio Lopez Luna, Felix T. Haase, and Beatriz Roldan Cuenya**

anie_202017070_sm_miscellaneous_information.pdf

Author Contributions

A.H. Data curation: Lead; Formal analysis: Lead; Methodology: Lead; Visualization: Lead; Writing – original draft: Lead; Writing – review & editing: Supporting
A.B. Data curation: Supporting; Formal analysis: Supporting; Methodology: Supporting; Supervision: Lead; Visualization: Supporting; Writing – review & editing: Supporting
H.J. Data curation: Supporting; Formal analysis: Supporting; Methodology: Supporting; Visualization: Supporting; Writing – review & editing: Supporting
J.T. Data curation: Supporting; Formal analysis: Supporting; Methodology: Supporting; Writing – review & editing: Supporting
S.K. Data curation: Supporting; Formal analysis: Supporting; Writing – review & editing: Supporting
C.R. Data curation: Supporting; Formal analysis: Supporting; Methodology: Supporting; Writing – review & editing: Supporting
M.L. Data curation: Supporting; Formal analysis: Supporting; Methodology: Supporting; Writing – review & editing: Supporting
F.H. Data curation: Supporting; Writing – review & editing: Supporting
B.R. Conceptualization: Lead; Formal analysis: Supporting; Funding acquisition: Lead; Methodology: Supporting; Supervision: Lead; Visualization: Supporting; Writing – review & editing: Lead.

Synthesis of Catalysts and Preparation of Electrodes

Cu_2O nanocubes (NCs) were prepared by a ligand-free method similar to that used in a prior work[1] and subsequently decorated with Ag nanoparticles (NPs). All reagents were purchased from Sigma-Aldrich in ACS grade and used without further purification. In a typical synthesis, an alkaline diluted solution of $CuCl_2$ was-prepared by adding 5 mL of a $CuCl_2$ * 2 H_2O solution (0.1 M) and 15 mL of a NaOH solution (0.2 M) to 200 mL of ultrapure water (> 18 MΩ cm^{-1}) at room temperature. After stirring for 5 min, 10 mL of a L-ascorbic acid solution (0.1 M) were added to the mixture. The solution was further stirred for 1 h. Then, 6 or 10 mL of an $AgNO_3$ solution (0.0025 M) were slowly added to the reaction solution under vigorous stirring and were further stirred for 10 min to obtain 3 and 5 at% Ag/Cu_2O NCs (denoted as 3-Ag/Cu_2O and 5-Ag/Cu_2O), respectively. The solution was centrifuged and washed three times, twice with an ethanol-water mixture (1:1) and once with pure ethanol. The final product was dispersed in 10 mL of ethanol to reach a Cu loading of 2 mg mL^{-1}. The solutions were stored in the fridge.

To prepare the electrode, 475 µL of the catalyst dispersion with a Cu loading of 2 mg mL^{-1} were mixed with 25 µL of a Nafion solution (Sigma-Aldrich, ~ 5 wt% in a mixture of alcohols and water) and then ultrasonicated for 5 min. Then, 37 µL of the catalyst mixture were slowly drop-casted on each side of a 0.5 x 2 cm carbon paper sheet (Alfa Aesar, Toray Carbon Paper, TGP-H-60) to obtain a Cu loading of 70 µg cm^{-2}. After drying, the electrode was ready to use in an H-type electrochemical cell for CO_2RR.

The reference sample Cu_2O/Ag was prepared by drop-casting the Cu_2O NCs on a mechanically polished Ag foil (Alfa Aesar, 0.1 mm thick, 99.998%) to obtain a Cu loading of 70 µg cm^{-2}.

Electrochemical Characterization

Electrochemical CO_2RR experiments were performed with a Biologic SP-300 potentiostat in a gas-tight custom-made H-type cell. The cathodic and anodic compartments were separated by an anion exchange membrane (Selemion AMV, AGC Inc.). We used a platinum gauze (MaTecK, 3600 mesh cm^{-2}) as the counter electrode and a reversible hydrogen electrode (RHE, HydroFlex, Gaskatel) as the reference electrode. Both compartments were filled with a 0.1 M $KHCO_3$ solution (Sigma-Aldrich, 99.7%). The electrolyte was previously purified from trace metal ion impurities by a cation-exchange resin (Chelex 100 Resin, Bio-Rad) followed by saturation with CO_2 (99.995%) for 30 min until a pH of 6.8 was reached. The electrochemical protocol consisted of a linear sweep voltammogram (LSV) from the open circuit potential to the cathodic potential followed by chronoamperometry (CA) at this potential for 2 h. All potentials are given versus the RHE scale and were corrected for the iR drop. Each presented data point at one potential corresponds to an average of at least three different

measurements collected with an identical freshly prepared sample under the same experimental conditions, and the error bars represent the standard deviation.

The gas products were measured every 15 min and quantified by online gas chromatography (GC, Agilent 7890B) equipped with a thermal conductivity detector (TCD) and a flame ionization detector (FID). During the measurements, CO_2 gas was constantly bubbled through the electrolyte with a flow rate of 20 mL min^{-1}. Carboxylates were analyzed with a high-performance liquid chromatograph (HPLC, Shimadzu Prominence) equipped with a NUCLEOGEL SUGAR 810 column and a refractive index detector (RID). Alcohols were quantified with a liquid GC (L-GC, Shimadzu 2010 plus) equipped with a fused silica capillary column and an FID detector. An aliquot of the electrolyte was collected after each measurement and directly analyzed by the HPLC and L-GC.

The catalyst surface roughness factors were estimated by measuring the double-layer capacitance with cyclic voltammetry in a non-Faradaic potential range from +0.10 to +0.25 V_{RHE} at scan rates of 20, 40, 60, 80, and 100 mV s^{-1} in a CO_2-saturated 0.1 M $KHCO_3$ solution after 2 h of electrochemical reaction at −1.0 V_{RHE}.[2]

Ex Situ Characterization.

The morphology and elemental distribution of the samples were determined by scanning transmission electron microscopy (STEM, FEI Talos F200X microscope, Thermo Fisher Scientific) and energy dispersive spectroscopy (EDX, SuperX 4 SDD EDX detector) maps. The STEM was equipped with an XFEG field emission gun (200 kV) and with brightfield (BF), darkfield (DF) and high angle annular darkfield (HAADF) detectors. Samples were prepared by coating a nickel grid (400 mesh with lacey carbon film, PLANO GmbH) with the catalyst dispersed in EtOH:H_2O (1:1) before and after CO_2RR.

The crystal structure of the catalysts was analyzed *ex situ* with an X-ray diffractometer (XRD, Bruker D8 Advance) equipped with an energy-dispersive position-sensitive LynxEye detector and Cu X-ray tube. Measurements of the as-prepared samples deposited on a low-background Si substrate were performed in a 2θ range of 10-90° with a step size of 0.03° and a collection time of 5 s per step in the Bragg-Brentano geometry. Grazing incident (GI) XRD measurements of the samples deposited on carbon paper before and after CO_2RR were performed in a 2θ range of 10-87° with a step size of 0.04°, a collection time of 26 s per step and an incident angle of 0.2°. For the grazing-incidence measurements, the XRD was equipped with a Goebel mirror parallelizing the X-ray beam, an equatorial Soller slit (0.3°) and the LynxEye detector in 0D mode. Rietveld refinement was applied to analyze the XRD pattern using the TOPAS® software package. Instrumental broadening and zero error were considered.

The bulk composition and amount of Cu and Ag in the catalyst dispersions and in the catalysts on carbon paper in the as-prepared samples and after CO_2RR were determined by inductively coupled

plasma-mass spectrometry (ICP-MS, iCAP RQ, Thermo Fisher Scientific). Samples were digested in a mixture of concentrated acids (HNO_3:H_2SO_4:HCl in a 1:1:3 ratio) and heated using microwave irradiation at 180 °C for 20 min, with a ramping step of 10 min (Multiwave GO, Anton Paar). The samples were diluted with ultrapure water (> 18 MΩ cm^{-1}) to reach the appropriate concentrations.

The surface composition of the catalysts was characterized by quasi-*in situ* X-ray photoelectron spectroscopy (XPS) before and directly after CO_2RR without exposure to air. For this purpose, an electrochemical cell was directly connected to an ultra-high vacuum system equipped with a hemispherical electron analyzer (Phoibos 100, SPECS GmbH, E_{pass} = 20 eV) and an X-ray source (XR 50, SPECS GmbH) with a Magnesium anode (1253.6 keV, 250 W). All spectra were aligned to the Cu $2p_{3/2}$ peak (E_{bin} = 932.7 eV). Linear combination fitting of the Cu LMM Auger profiles using reference spectra was performed to distinguish the different copper oxidation states. The Cu, Cu_2O and CuO amounts were estimated from the integrated areas of the fit with the corresponding reference spectra. These reference spectra were acquired on an *in situ* annealed and sputtered Cu foil (metallic reference) and a plasma oxidized CuO sample, while the reference spectrum of Cu_2O was taken from the literature.[3] The Cu to Ag ratio was determined from the Ag $3d_{5/2}$ to Cu $2p_{3/2}$ regions and calculated considering the relative sensitivity factors (RSF) of the metals (Ag $3d_{5/2}$: 18.04; Cu $2p_{3/2}$: 15.9). The electrochemical measurements were performed using a Metrohm Autolab potentiostat (PGSTAT 302 N) with a platinum mesh as counter electrode and a leak-free Ag/AgCl as reference electrode.

Operando Characterization.

Operando X-ray absorption spectroscopy (XAS) measurements were performed at the SAMBA beamline at SOLEIL synchrotron in France (for Cu_2O NCs), and at the CLAESS beamline at ALBA synchrotron in Spain (for 3-Ag/Cu_2O and 5-Ag/Cu_2O samples). More details on the XAS data collection and EXAFS data fitting can be found below.

Operando surface-enhanced Raman spectroscopy (SERS) was carried out with a Raman spectrometer (Renishaw, InVia Reflex) coupled with an optical microscope (Leica Microsystems, DM2500M) together with a motorized stage for sample tracking (Renishaw, MS300 encoded stage). Calibration of the system was performed using a Si(100) wafer (520.5 cm^{-1}). A near-infrared laser (Renishaw, RL785, λ = 785 nm, P_{max} = 500 mW) served as excitation sources. Backscattered light was Rayleigh-filtered and the Raman scattering was collected in the range of 100-3200 cm^{-1} with a grating of 1200 lines mm^{-1} and directed to a CCD detector (Renishaw, Centrus). For the *operando* measurements, the excitation source was focused on the surface of the sample and Raman scattering signals were collected with a water immersion objective (Leica microsystems, 63x, numerical aperture of 0.9). The water immersion objective was protected from the electrolyte by a Teflon film (DuPont, film thickness of 0.013 mm) wrapped around the objective. The electrochemical measurements were

performed in a home-built spectro-electrochemical cell made of PTFE and controlled by a Biologic SP-240 potentiostat. The cell was equipped with a leak-free Ag/AgCl reference electrode and a Pt counter electrode, while the working electrode with the catalyst was drop-casted on glassy carbon. A CO_2-saturated 0.1 M $KHCO_3$ solution was used as electrolyte. Each potential was applied for at least 10 min before collecting the spectra to ensure steady-state conditions at the surface of the catalyst. The collection of each spectrum was performed with 10 s of exposure time. Depth scans were performed to optimize the focus in order to increase the Raman signal of the species studied. Bands at higher wavenumbers (~3000 cm^{-1}) typical for hydrocarbons were not accessible under infrared laser excitation. The Raman data were processed using the Renishaw WiRE 5.2 software. The spectra were baseline-subtracted using the intelligent spline feature (polynomial order 8) and cosmic rays were removed.

Details of XAS Data Collection and Analysis.

Operando Cu K-edge XAS measurements of Cu_2O NCs were measured using a Si(220) monochromator for energy selection. Higher harmonics were rejected using a Pd-coated mirror. The beam size was 1 x 0.5 mm. Measurements were performed in fluorescence mode using a 13-channel Ge detector. The intensity of the incident radiation was measured with an ionization chamber (I_0) filled with an N_2(500 mbar)/He(500 mbar) mixture. Two additional ionization chambers filled with 1700 mbar N_2 (in I_1 chamber) and an Ar(150 mbar)/N_2(850 mbar) mixture (in I_2 chamber) were used for measurements in transmission mode for reference samples.

Operando XAS measurements for 3-Ag/Cu_2O and 5-Ag/Cu_2O were performed in a similar manner using a 6-channel Si drift detector for fluorescence data collection. A Si(111) monochromator was used for measurements at the Cu K-edge, and a Si(311) monochromator for measurements at the Ag K-edge. In the latter case, the I_0 chamber was filled with 93:7 Kr to N_2 mixture. For bimetallic samples, XAS spectra for the Cu K-edge (E_0 = 8979 eV) and Ag K-edge (E_0 = 25514 eV) were collected in fluorescence mode separately, using two fresh samples with different sample loadings to ensure reasonable XAS data quality for more diluted Ag but avoid self-absorption effects in the Cu K-edge spectra.

A custom-built electrochemical cell was used for *operando* XAS measurements. The applied potential was controlled by a PalmSens potentiostat (MultiEmStat). A platinum mesh and leak free Ag/AgCl electrode were used as counter and reference electrodes, respectively. For the XAS studies, a sample was drop-casted onto one side of a carbon paper (Sigracet 29 BC, SGL Carbon) substrate (with a loading of 0.5 mg cm^{-2} for the Cu K-edge measurements and 3 mg cm^{-2} for Ag K-edge measurements), while the other side of the carbon paper was covered with Kapton tape. Subsequently, the sample was mounted in the *operando* cell with the Kapton side facing out of the cell and acting as a window, while

the side coated with the catalyst was in contact with the electrolyte. The electrolyte (0.1 M $KHCO_3$) was circulated between the cell and a reservoir using a peristaltic pump. The cell was continuously purged with CO_2 during the measurements. All measurements were performed under a constant potential of −1.0 V_{RHE}.

Time-resolved spectra under CO_2RR conditions were acquired with 9-12 min acquisition time per spectrum until no further changes were observed. For all samples, several hours were needed to achieve a steady-state structure and composition. Analysis of the time-dependent X-ray absorption near edge structure (XANES) and extended X-ray absorption fine structure (EXAFS) spectroscopy data was used then to follow the evolution of the chemical composition and the sample structure at the Cu K-edge. At the Ag K-edge, the analysis of individual XAS spectra collected *operando* under CO_2RR conditions was not possible due to the low concentration of Ag. Therefore, the Ag K-edge XAS spectra obtained during the first 2 h under CO_2RR conditions were merged together and compared with those for the as-prepared sample in air. Linear combination fitting (LCF) was applied to process the XANES data. Moreover, Cu- and Ag K-edge EXAFS data fitting was performed to gain quantitative information about the bond lengths and coordination numbers.

Data alignment and normalization of the XANES data were carried out using the conventional approach implemented in the Athena software.[4] For quantitative EXAFS analysis, conventional least-square fitting to theoretical standards, as implemented in the FEFFIT code[4] was applied. Theoretical phases and amplitudes were obtained in self-consistent ab-initio calculations with the FEFF8.5 code.[5] The complex exchange-correlation Hedin-Lundqvist potential and default values of muffin-tin radii as provided within the FEFF8.5 code were employed. Fitting of the Cu K-edge EXAFS spectra $\chi(k)k^2$ of the as-prepared catalysts was carried out in *R*-space in the range from R_{min} = 1 Å up to R_{max} = 2.1 Å, while for the catalysts in the reduced state, R_{min} = 1.0 Å to R_{max} = 3.0 Å were used. The Fourier transforms were carried out in the *k*-range from 3.0 Å^{-1} to 10.0 Å^{-1} with a *k*-weighting of 1, 2 and 3. Fitting of Ag K-edge EXAFS spectra of the as-prepared catalysts and the catalysts during CO_2RR was carried out in *R*-space in the range from R_{min} = 1.4 Å up to R_{max} = 3.2 Å. The Fourier transforms were carried out in the *k*-range from 3.0 Å^{-1} up to 8.0 Å^{-1} with a *k*-weighting of 1,2 and 3. Fitting parameters were the coordination numbers *N*, interatomic distances *R*, disorder factors σ^2 for Cu-O, Cu-Cu, Ag-Ag and Ag-Cu paths, as well as the corrections to the photoelectron reference energies ΔE_0. The S_0^2 factors were obtained from the EXAFs fit data of the references, which were 0.87 ± 0.02 for Cu foil, 0.32 ± 0.01 for Cu_2O and 0.92 ± 0.02 for Ag foil.

Supporting Figures and Tables.

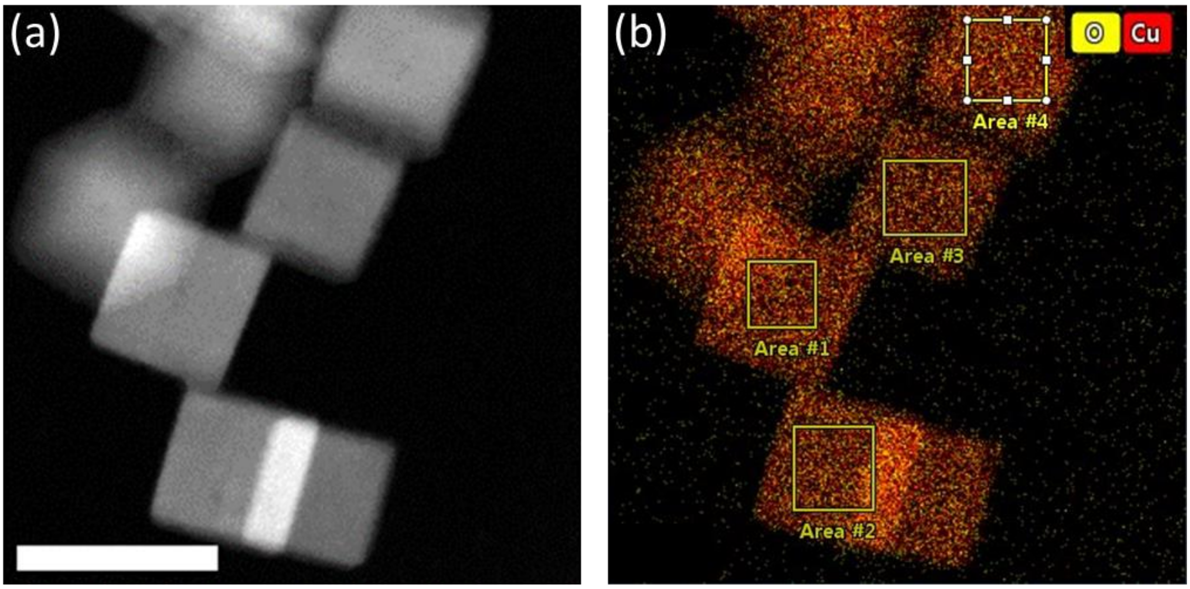

Figure S1. STEM-HAADF image (a) with corresponding EDX map (b) of an as-prepared Cu_2O NC sample. The STEM-EDX image shows four selected areas to determine the Cu:O atomic ratio. The scale bar corresponds to 50 nm.

Table S1. Cu:O atomic ratio of as-prepared Cu_2O NCs obtained from the STEM-EDX maps in Figure S1.

Area [#]	Cu [at%]	O [at%]
**1**	67(15)	33(6)
**2**	65(15)	35(7)
**3**	65(15)	35(7)
**4**	67(16)	33(6)

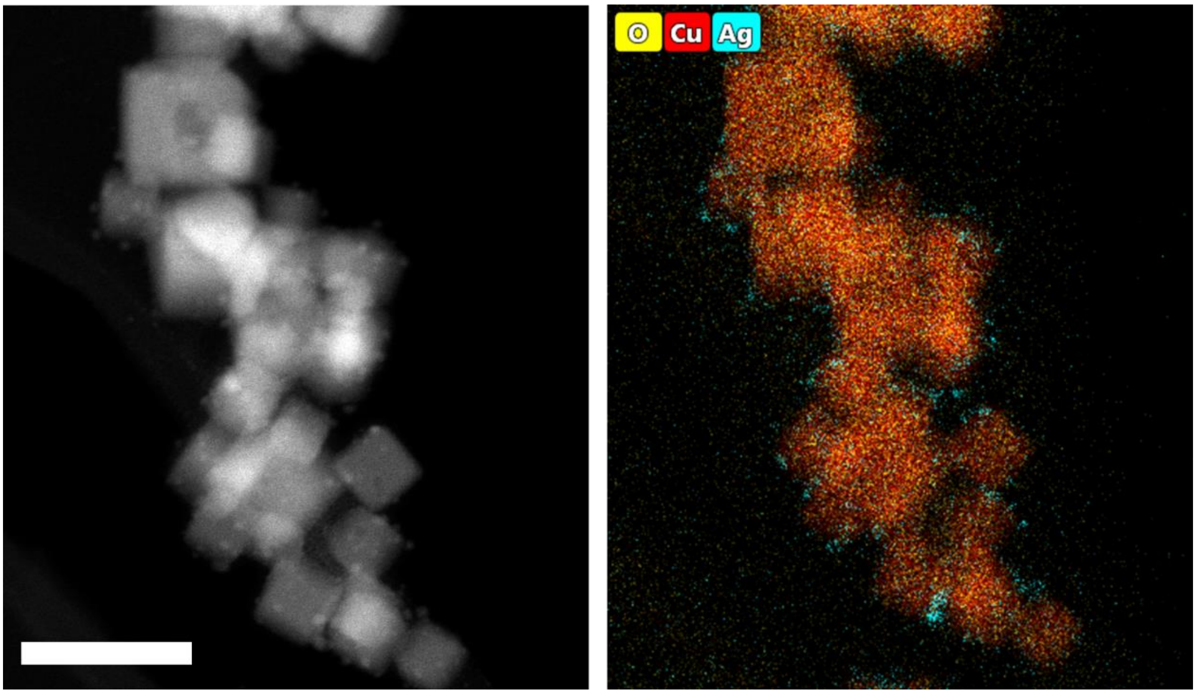

Figure S2. STEM-HAADF image (left) with corresponding EDX map (right) of an as-prepared 3-Ag/Cu_2O sample. The scale bar corresponds to 100 nm.

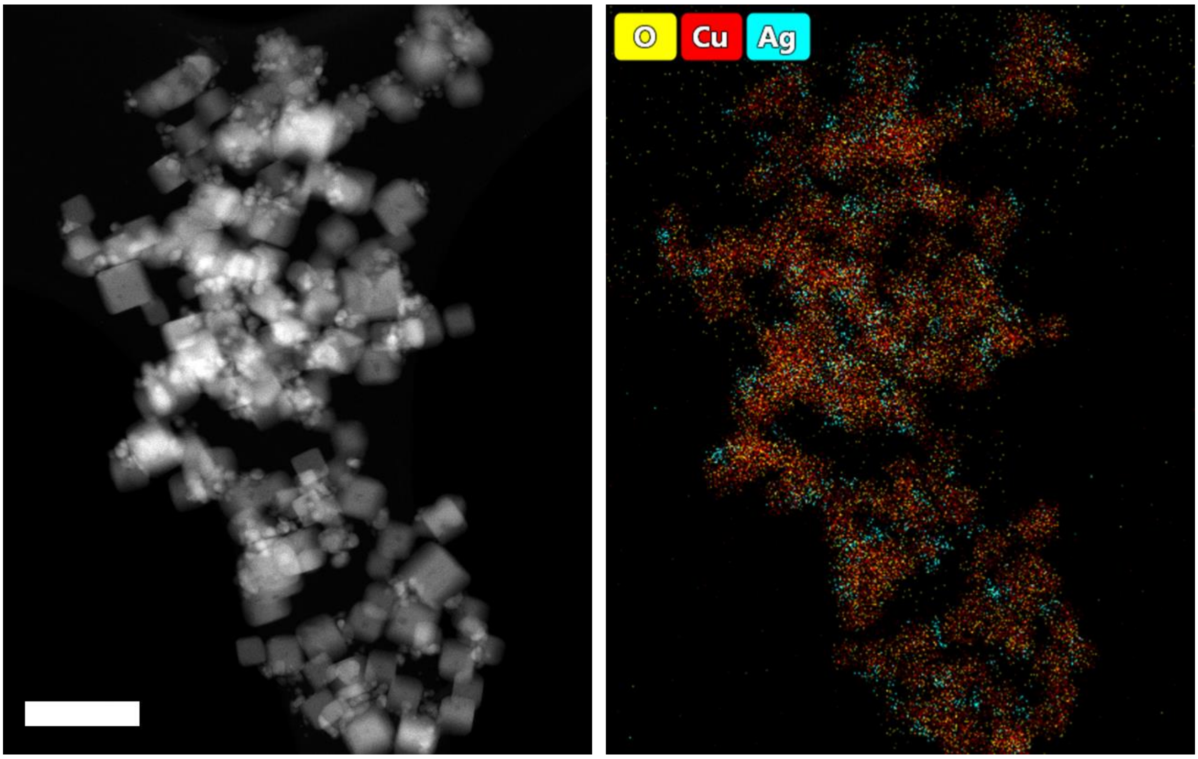

Figure S3. STEM-HAADF image (left) with corresponding EDX map (right) of an as-prepared 5-Ag/Cu_2O sample. The scale bar corresponds to 100 nm.

Table S2. Edge lengths of Cu_2O NCs and diameters of Ag NPs with the corresponding size distribution obtained from the analysis of STEM-HAADF images of Cu_2O NCs, 3-Ag/Cu_2O and 5-Ag/Cu_2O in the as-prepared state and after 2 h of CO_2RR at -1.0 V_{RHE}. The decrease in the edge length after CO_2RR was slightly enhanced when the Cu_2O NCs were decorated with Ag NPs.

**Sample**	**As-prepared**		**After CO_2RR**	
	**Cu_2O NCs [nm]**	**Ag NPs [nm]**	**Cu_2O NCs [nm]**	**Ag NPs [nm]**
**Cu_2O NCs**	34.8 ± 6.6	-	32.5 ± 9.6	-
**3-Ag/Cu_2O**	37.0 ± 8.3	4.6 ± 1.1	32.8 ± 10.5	6.3 ± 1.6
**5-Ag/Cu_2O**	27.6 ± 4.2	6.0 ± 2.1	24.2 ± 7.4	9.5 ± 2.5

Table S3. Elemental quantification of Cu, O and Ag extracted from the STEM-EDX images of Figure 1 of Cu_2O NCs, 3-Ag/Cu_2O and 5-Ag/Cu_2O.

**Sample**	**As-prepared**			**After CO_2RR**		
	**Cu [%]**	**O [%]**	**Ag [%]**	**Cu [%]**	**O [%]**	**Ag [%]**
**Cu_2O NCs**	66(15)	34(6)	-	79(19)	21(4)	-
**3-Ag/Cu_2O**	59(13)	40(7)	1.4(3)	76(18)	23(5)	0.6(1)
**5-Ag/Cu_2O**	61(13)	36(6)	3.4(7)	65(14)	31(6)	3.9(8)

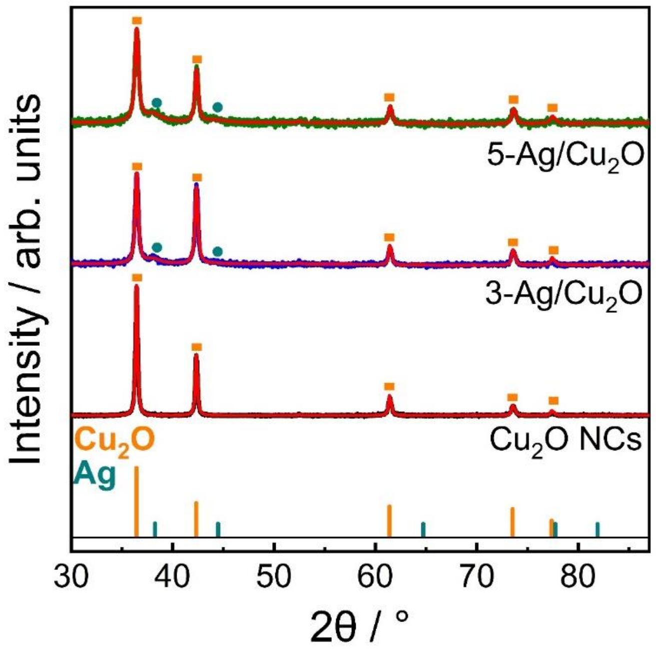

Figure S4. *Ex situ* XRD pattern of as-prepared Cu_2O NCs (black), 3-Ag/Cu_2O (blue) and 5-Ag/Cu_2O (green) samples on a Si substrate with corresponding Rietveld fits (red). The intensity ratio of the *fcc* Cu (111) and (200) reflections varies most likely due to a preferred orientation of the Cu_2O NCs on the Si substrate. No further reflections, e.g. from CuO, metallic Cu, AgCl or Ag_2O can be found in the as-prepared state, confirming the phase purity of the catalysts.

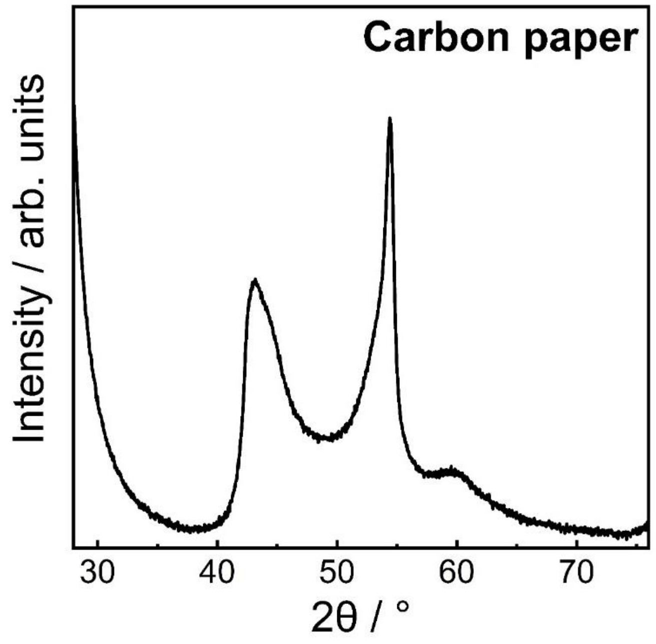

Figure S5. *Ex situ* grazing incidence XRD pattern of the carbon paper support.

Table S4. Lattice parameters extracted from Rietveld refinement of *ex situ* XRD pattern of as-prepared Cu_2O NCs, 3-Ag/Cu_2O and 5-Ag/Cu_2O samples; and of 5-Ag/Cu_2O deposited on carbon paper after 2 h of CO_2RR at -1.0 V_{RHE}. Comparison to literature values of the lattice parameters of Cu_2O, Cu and Ag bulk is given below.

**Sample**	**Lattice parameters [Å]**		
	**Cu_2O**	***fcc* Cu**	***fcc* Ag**
As-prepared			
**Cu_2O NC**	4.2656(19)	-	-
**3-Ag/Cu_2O**	4.2674(5)	-	4.094(4)
**5-Ag/Cu_2O**	4.2641(8)	-	4.092(3)
After CO_2RR			
**5-Ag/Cu_2O**	4.2653(9)	3.612(2)	4.088(3)
References			
**Cu_2O bulk**	4.27[6]	-	-
**Cu bulk**	-	3.597[7]	-
**Ag bulk**	-	-	4.079[7]

Table S5. Cu and Ag composition of as-prepared Cu_2O NCs, 3-Ag/Cu_2O and 5-Ag/Cu_2O dispersions obtained by ICP-MS.

Sample	Cu [at%]	Ag [at%]
**Cu_2O NCs**	100	-
**3-Ag/Cu_2O**	97.75(1)	2.25(1)
**5-Ag/Cu_2O**	95.07(3)	4.93(3)

Table S6. Cu and Ag mass and composition of Cu_2O NCs, 3-Ag/Cu_2O and 5-Ag/Cu_2O on carbon paper (2 cm^2) obtained by ICP-MS in as-prepared state and after 2 h of CO_2RR at -1.0 V_{RHE}.

Sample	As-prepared				After CO_2RR			
	Cu [µg]	Ag [µg]	Cu [at%]	Ag [at%]	Cu [µg]	Ag [µg]	Cu [at%]	Ag [at%]
**Cu_2O NCs**	139(32)	-	100	-	134(7)	-	100	-
**5-Ag/Cu_2O**	146(14)	12(1)	95.3(2)	4.7(2)	149(17)	12(2)	95.3(2)	4.7(2)

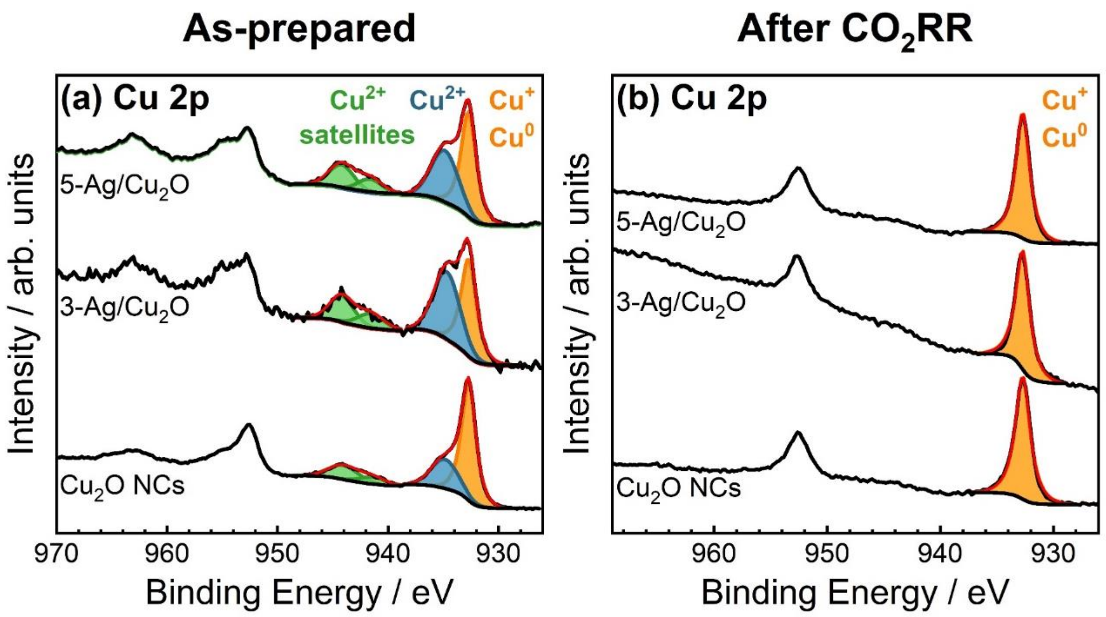

Figure S6. Quasi-*in situ* Cu 2p core level regions of Cu_2O NCs, 3-Ag/Cu_2O and 5-Ag/Cu_2O (a) in as-prepared state and (b) after 2 h of CO_2RR at -1.0 V_{RHE} (without air exposure) with corresponding fits (red line). The Cu 2p core level regions of the as-prepared samples show the presence of Cu(0)/Cu(I) and a shake-up satellite corresponding to Cu(II).

Table S7. Kinetic energies and composition of Cu_2O, CuO and Cu for Cu_2O NCs, 3-Ag/Cu_2O and 5-Ag/Cu_2O in as-prepared state and after 2 h CO_2RR obtained by integration of the Cu LMM quasi-in situ XPS spectra shown in Figures 3c and 3d. The presence of surface CuO can explain the discrepancy between STEM and XRD particle sizes, because of the possible amorphous character of CuO.

**Sample**	**As-prepared**				**After CO_2RR**			
	**Cu_2O**		**CuO**		**Cu_2O**		**Cu**	
	**Peak [eV]**	**Fraction [at%]**	**Peak [eV]**	**Fraction [at%]**	**Peak [eV]**	**Fraction [at%]**	**Peak [eV]**	**Fraction [at%]**
**Cu_2O NCs**	916.1	84	917.2	16	916.4	5	918.5	95
**3-Ag/Cu_2O**	916.1	77	917.2	23	916.4	0	9.18.5	100
**5-Ag/Cu_2O**	916.1	77	917.2	23	916.4	2	918.5	98

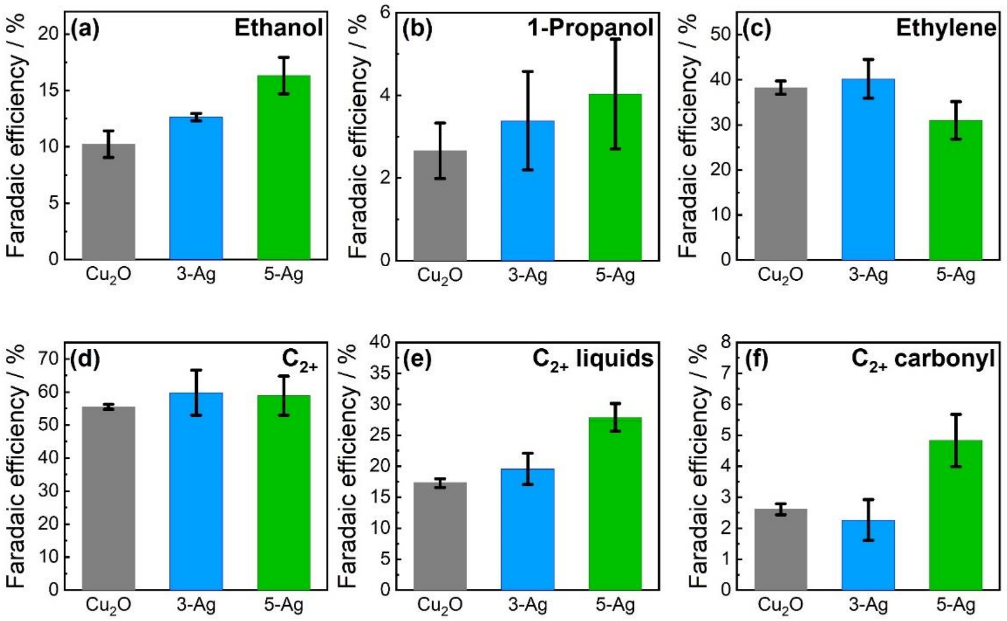

Figure S7. Faradaic efficiencies after 2 h electrolysis at -1.0 V_{RHE} of (a) ethanol, (b) 1-propanol, (c) ethylene, (d) C_{2+} products, (e) C_{2+} liquid products and (f) C_{2+} carbonyl products of Cu_2O NCs (gray), 3-Ag/Cu_2O (blue) and 5-Ag/Cu_2O (green) in CO_2-saturated 0.1 M $KHCO_3$.

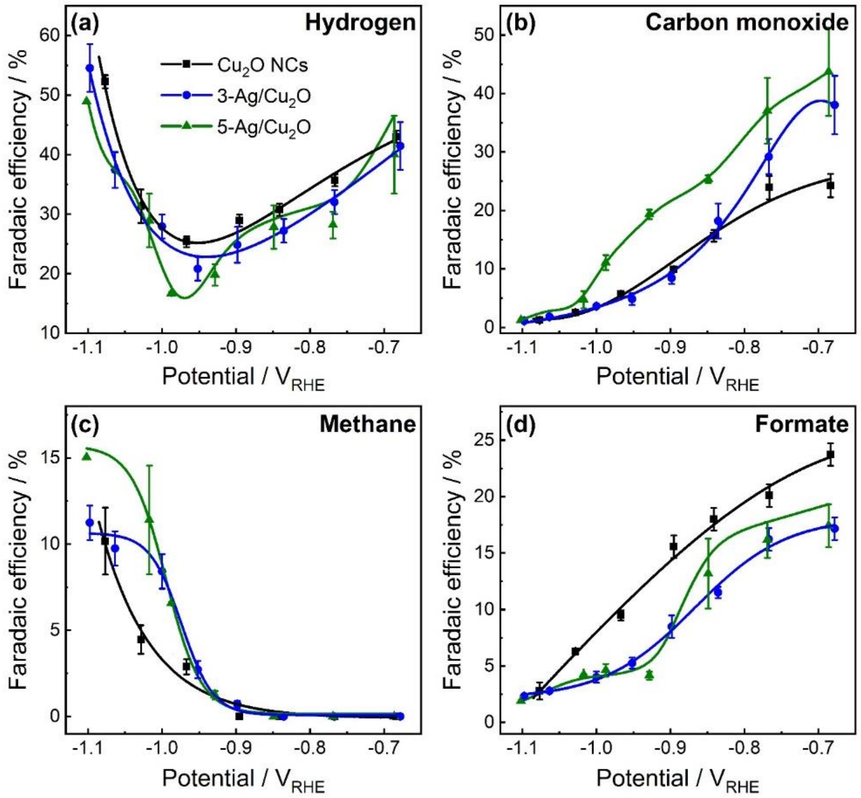

Figure S8. Potential-dependent Faradaic efficiencies of the major C_1 products (a) hydrogen, (b) carbon monoxide, (c) methane and (d) formate of Cu_2O NCs (black), 3-Ag/Cu_2O (blue) and 5-Ag/Cu_2O (green) samples after 2 h of electrolysis in CO_2-saturated 0.1 M $KHCO_3$. Solid lines are a guide for the eye.

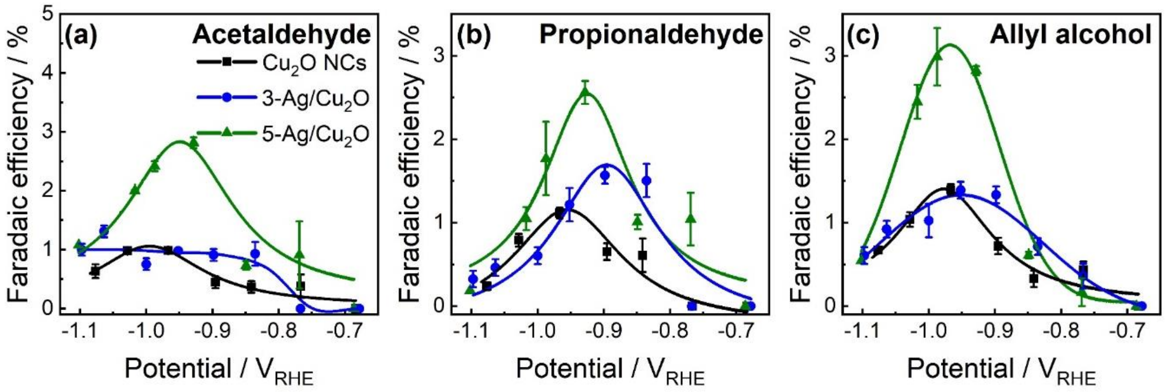

Figure S9. Potential-dependent Faradaic efficiencies of the minor C_{2+} products (a) acetaldehyde, (b) propionaldehyde and (c) allyl alcohol of Cu_2O NCs (black), 3-Ag/Cu_2O (blue) and 5-Ag/Cu_2O (green) after 2 h of electrolysis in CO_2-saturated 0.1 M $KHCO_3$. Solid lines are a guide for the eye.

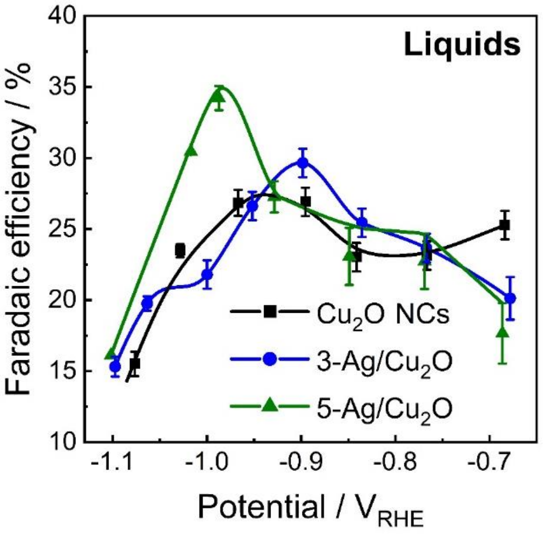

Figure S10. Potential-dependent combined Faradaic efficiency of the sum of the liquid products (including formate, acetate, acetaldehyde, propionaldehyde, acetone, ethanol, 1-propanol and allyl alcohol) of Cu_2O NCs (black), 3-Ag/Cu_2O (blue) and 5-Ag/Cu_2O (green) after 2 h of electrolysis in CO_2-saturated 0.1 M $KHCO_3$. Solid lines are a guide for the eye.

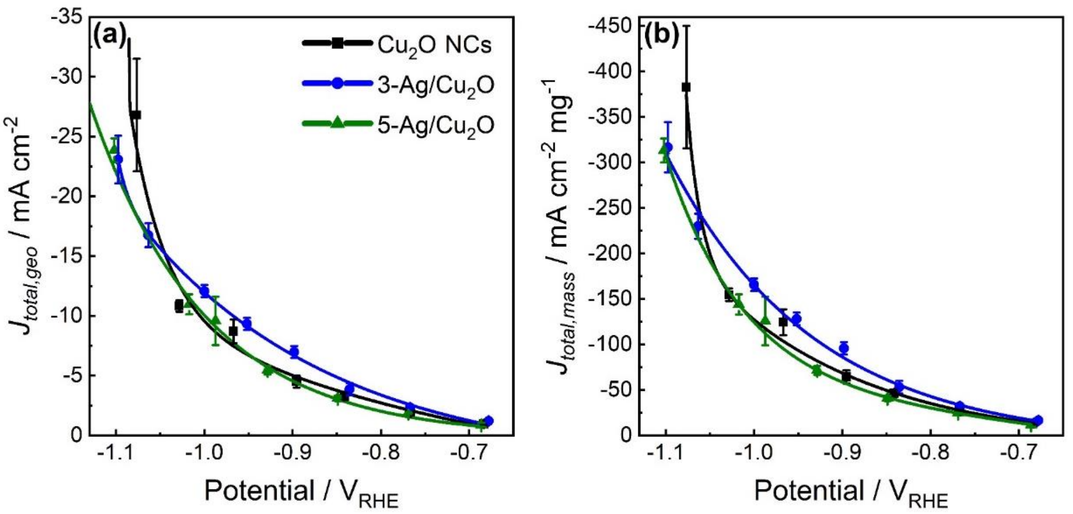

Figure S11. (a) Potential-dependent current densities normalized to the geometric area of the electrode ($J_{total,geo}$) and (b) to the deposited mass of Cu and Ag ($J_{total,mass}$) of Cu_2O NCs (black), 3-Ag/Cu_2O (blue) and 5-Ag/Cu_2O (green) in CO_2-saturated 0.1 M $KHCO_3$. The geometric and mass current densities of the samples are similar, suggesting similar total activities. The reported values represent the average currents of 2 h of electrolysis . Solid lines are a guide for the eye.

Table S8. Capacitance values of the catalysts and references determined by electrochemical double layer capacitance measurements acquired directly after 2 h of CO_2RR at −1.0 V_{RHE}.

Sample	Capacitance [mF cm^{-2}]
Catalysts	
**Cu_2O NCs**	0.15(2)
**3-Ag/Cu_2O**	0.27(2)
**5-Ag/Cu_2O**	0.40(2)
References	
**Cu foil**	0.027[8]
**Ag foil**	0.042[9]
**Carbon paper**	0.05(1)

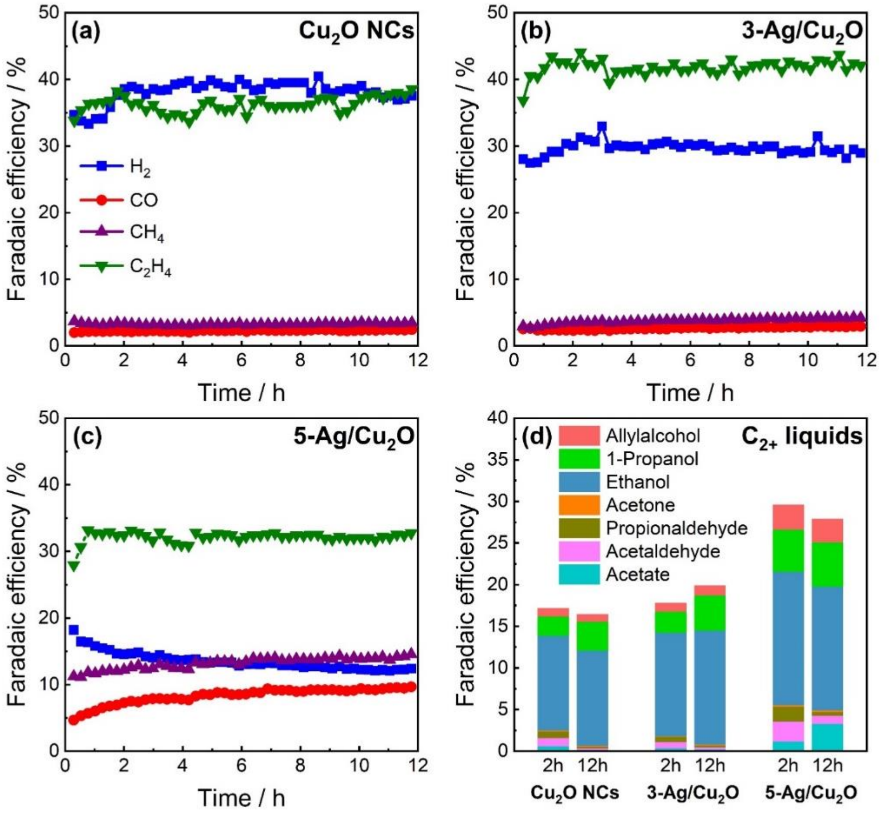

Figure S12. Stability measurements during 12 h of CO_2RR at -1.0 V_{RHE} in CO_2-saturated 0.1 M $KHCO_3$. Time-dependent Faradaic efficiencies of the gaseous products of (a) Cu_2O NCs, (b) 3-Ag/Cu_2O and (c) 5-Ag/Cu_2O. (d) Corresponding evolution of the C_{2+} liquid products after 2 h and after 12 h of CO_2RR of the different samples. For the Ag/Cu_2O samples, the production of CO and CH_4 rises, while H_2 is further suppressed. The amount of C_{2+} liquid products over time remains stable. It should be noted that the liquid products could not be measured online and therefore, only data after 2 h and 12 h are available.

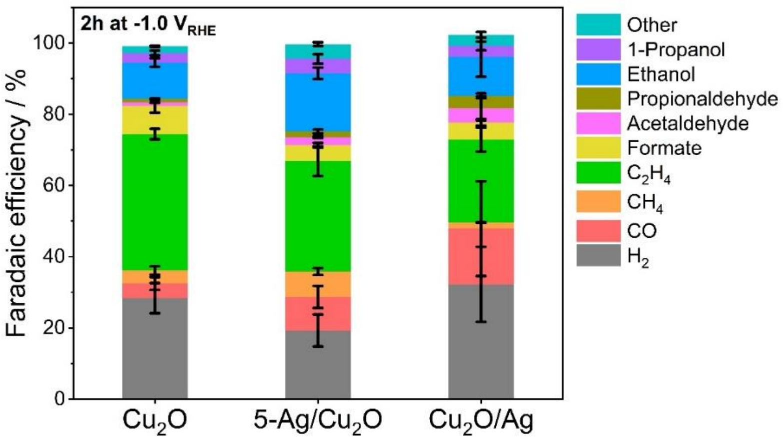

Figure S13. Faradaic efficiencies of Cu_2O NCs, 5-Ag/Cu_2O (5 at% Ag NPs-decorated Cu_2O NCs) and Cu_2O/Ag (Cu_2O NCs drop-casted on an Ag foil) after 2 h of CO_2RR at -1.0 V_{RHE} in CO_2-saturated 0.1 M $KHCO_3$. Products indicated as other are acetate, acetone, 2-propanol and allyl alcohol.

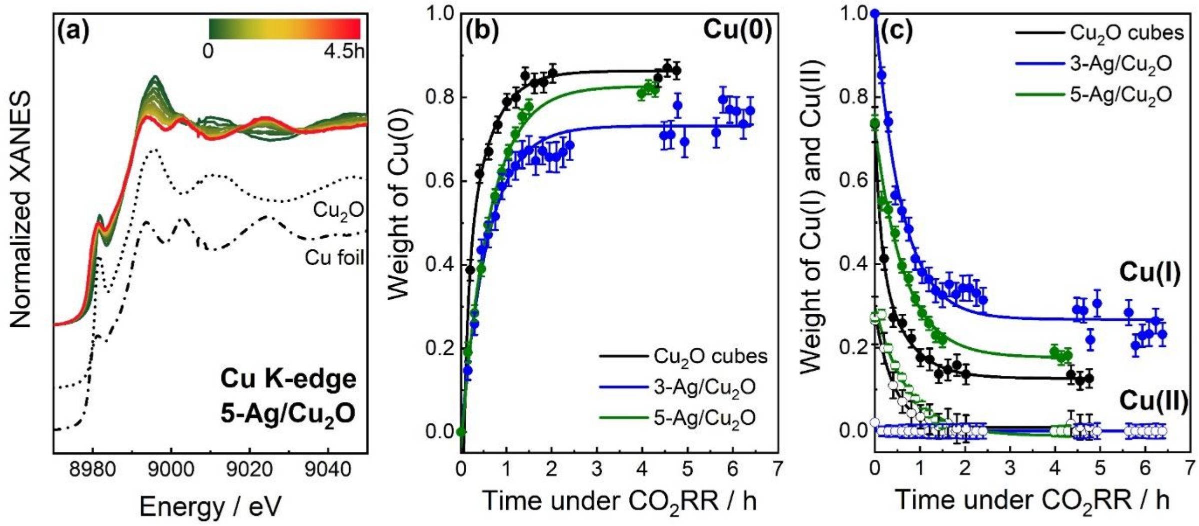

Figure S14. Temporal evolution of (a) the normalized Cu K-edge XANES spectra of 5-Ag/Cu_2O and of the weights of (b) Cu(0) and (c) Cu(I) (filled circles) and Cu(II) (open circles) contributions to the Cu K-edge XANES spectra of Cu_2O NCs (black), 3-Ag/Cu_2O (blue) and 5-Ag/Cu_2O (green) during CO_2RR at -1.0 V_{RHE}, as obtained from linear combination fits. The differences in Cu(II) content of the different catalysts could be related to an aging effect of the samples.

Table S9. Comparison of Cu(0) and Cu(I) concentrations, as determined by XANES and EXAFS analysis for Cu_2O NCs and Ag-decorated NCs during CO_2RR at -1.0 V_{RHE}. The ratios of the different Cu species could be obtained from the EXAFS analysis since the reduced Cu-Cu coordination number for NCs under reaction conditions is a result of sample-averaging effect, where the measured EXAFS signal is an average of contributions from metallic and oxidized phases. In fact, the deviation of the Cu-Cu coordination number values from the bulk values can be used to independently estimate the concentrations of Cu(0) and Cu(I) species (providing that size effects on the FT-EXAFS spectra are negligible).

**Sample**	**XANES**		**EXAFS**	
	**Cu(0) [%]**	**Cu(I) [%]**	**Cu(0) [%]**	**Cu(I) [%]**
**Cu_2O NCs**	86(2)	14(2)	80(12)	20(12)
**3-Ag/Cu_2O**	77(3)	23(3)	80(5)	20(5)
**5-Ag/Cu_2O**	82(2)	18(2)	83(5)	17(5)

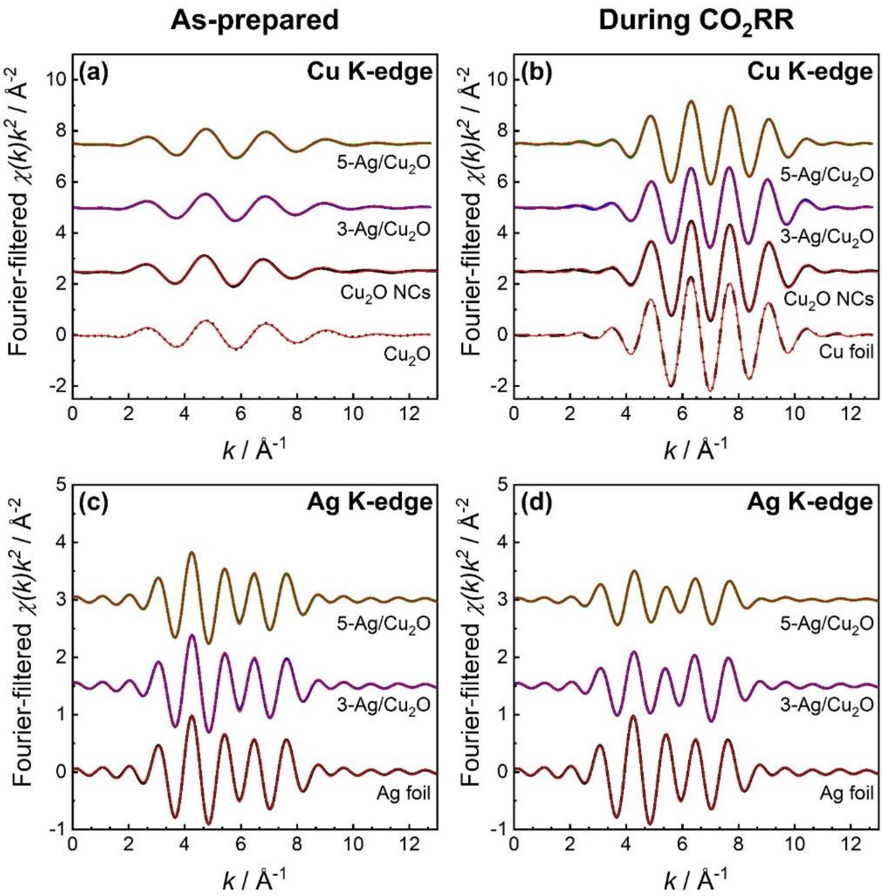

Figure S15. Fourier-filtered Cu and Ag K-edge EXAFS spectra in *k*-space of Cu_2O NCs (black), 3-Ag/Cu_2O (blue) and 5-Ag/Cu_2O (green) in as-prepared state (a, c) and in the final state under operando CO_2 reduction conditions at -1.0 V_{RHE} (b, d) with corresponding fits (red). Reference spectra of Cu_2O, Cu and Ag foils are shown for comparison.

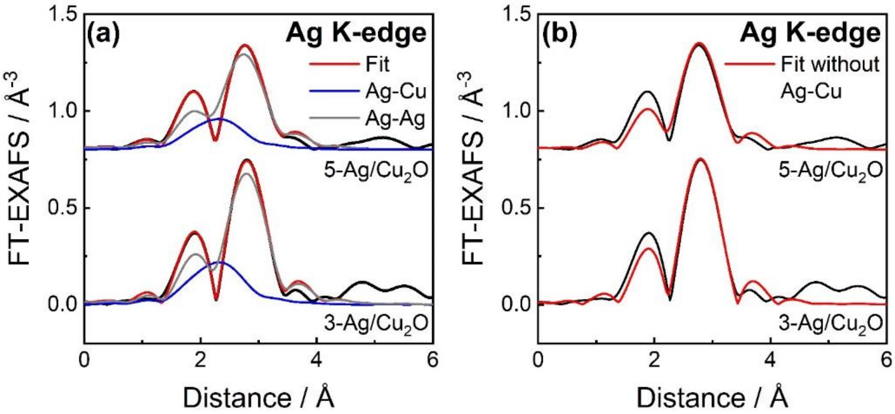

Figure S16. Moduli of the Fourier-transformed (FT) Ag K-edge EXAFS spectra of 3-Ag/Cu_2O and 5-Ag/Cu_2O in the final state under *operando* CO_2 reduction conditions at -1.0 V_{RHE}: (a) fits including a Ag-Cu contribution (red). Partial contribution of Ag-Ag (gray) and Ag-Cu bonds (blue) are also shown. Analogous fits but without the Ag-Cu contribution (red) are shown in (b).

Table S10. Structural parameters (coordination numbers *N*, interatomic distances *R* and disorder factors σ^2) obtained from the fit of experimental Cu K-edge EXAFS data acquired before and during CO_2RR at -1.0 V_{RHE}.

Sample	$N_{Cu\text{-}Cu}$	$R_{Cu\text{-}Cu}$ [Å]	$\sigma^2_{Cu\text{-}Cu}$ [Å^2]	$N_{Cu\text{-}O}$	$R_{Cu\text{-}O}$ [Å]	$\sigma^2_{Cu\text{-}O}$ [Å^2]	ΔE_0 [eV]	*R*- factor [%]
**Cu foil (Ref)**	12	2.527(2)	0.009(3)	0	-	.	2.5(3)	0.24
**Cu_2O (Ref)**	-	-	-	2	1.850(3)	0.0012(5)	1.4(5)	0.59
**Cu_2O NCs (as-prep)**	-	-	-	2.4(1)	1.900(6)	0.003(1)	1.5(7)	2.01
**3-Ag/Cu_2O (as-prep)**	-	-	-	1.8(1)	1.863(3)	0.0009(5)	1.4(4)	0.61
**5-Ag/Cu_2O (as-prep)**	-	-	-	2.0(1)	1.870(3)	0.0028(5)	0.7(4)	0.52
**Cu_2O NCs (-1.0 V_{RHE})**	9.4(4)	2.524(3)	0.0082(4)	0.4(3)	1.81(5)	0.01(2)	2.6(5)	0.56
**3-Ag/Cu_2O (-1.0 V_{RHE})**	7.4(3)	2.558(3)	0.0074(4)	0.3(1)	1.81(3)	0.001(7)	4.4(4)	0.53
**5-Ag/Cu_2O (-1.0 V_{RHE})**	8.7(3)	2.521(2)	0.0091(3)	0.3(1)	1.93(2)	0.002(7)	2.1(4)	0.36

Table S11. Structural parameters (coordination numbers *N*, interatomic distances *R* and disorder factors σ^2) obtained from the fit of experimental Ag K-edge EXAFS data collected before and during CO_2RR at -1.0 V_{RHE}, as well as corrections to photoelectron reference energies ΔE_0.

Sample	N_{Ag-Ag}	R_{Ag-Ag} [Å]	σ^2_{Ag-Ag} [Å^2]	N_{Ag-Cu}	R_{Ag-Cu} [Å]	σ^2_{Ag-Cu} [Å^2]	ΔE_0 [eV]	*R*-factor [%]
**Ag foil (Ref)**	12	2.833(3)	0.0116(4)	0	-	-	1.4(2)	0.17
**3-Ag/Cu_2O (as-prep)**	11.4(4)	2.829(3)	0.0130(5)	0	-	-	1.4(2)	0.33
**5-Ag/Cu_2O (as-prep)**	10.5(3)	2.831(3)	0.0126(5)	0	-	-	1.3(2)	0.35
**3-Ag/Cu_2O (-1.0 V_{RHE})**	9.0(3)	2.840(5)	0.0142(9)	1.1(2)	2.623(5)	0.003(3)	2.1(2)	0.07
**5-Ag/Cu_2O (-1.0 V_{RHE})**	8.5(6)	2.787(7)	0.019(2)	0.6(2)	2.596(8)	0.000(4)	0.8(4)	0.23

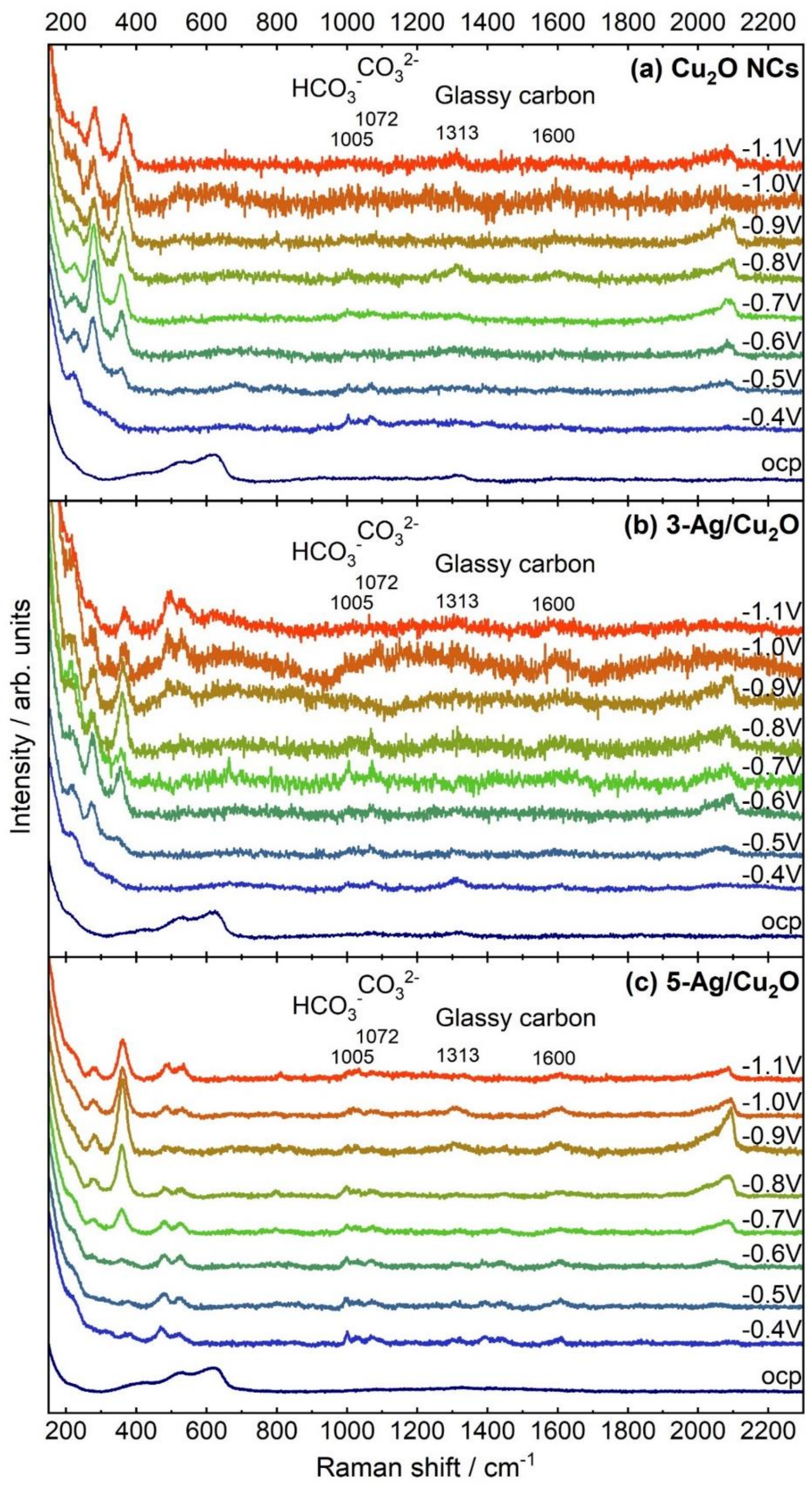

Figure S17. *Operando* surface-enhanced Raman spectra of (a) Cu_2O NCs, (b) 3-Ag/Cu_2O and (c) 5-Ag/Cu_2O at open circuit potential (ocp) and different applied potentials in CO_2-saturated 0.1 M $KHCO_3$. The potentials are given with reference to RHE. The characteristic peaks of Cu_2O are observed at 415 cm^{-1} (multiphonon process), at 527 cm^{-1} (Raman active F_{2g} mode), and at 624 cm^{-1} (IR active F_{1u} mode).[10] The peaks at 1005 and 1072 cm^{-1} can be assigned to the vibrations of HCO_3^- and CO_3^{2-} in aqueous solution, respectively, and the peaks at 1313 and 1600 cm^{-1} correspond to the glassy carbon support.[11] No peaks were detected from 2300 to 3000 cm^{-1}.

References

[1] X. W. Liu, F. Y. Wang, F. Zhen, J. R. Huang, *RSC Adv.* **2012**, *2*, 7647-7651.

[2] H. Mistry, A. S. Varela, C. S. Bonifacio, I. Zegkinoglou, I. Sinev, Y.-W. Choi, K. Kisslinger, E. A. Stach, J. C. Yang, P. Strasser, B. Roldan Cuenya, *Nat. Commun.* **2016**, *7*, 12123.

[3] M. C. Biesinger, *Surf. Interface Anal.* **2017**, *49*, 1325-1334.

[4] B. Ravel, M. Newville, *J. Synchrotron Radiat.* **2005**, *12*, 537-541.

[5] A. L. Ankudinov, B. Ravel, J. J. Rehr, S. D. Conradson, *Phys. Rev. B* **1998**, *58*, 7565-7576.

[6] A. Werner, H. D. Hochheimer, *Phys. Rev. B* **1982**, *25*, 5929-5934.

[7] W. P. Davey, *Phys. Rev.* **1925**, *25*, 753-761.

[8] J.-J. Velasco-Vélez, C.-H. Chuang, D. Gao, Q. Zhu, D. Ivanov, H. S. Jeon, R. Arrigo, R. V. Mom, E. Stotz, H.-L. Wu, T. E. Jones, B. Roldan Cuenya, A. Knop-Gericke, R. Schlögl, *ACS Catal.* **2020**, *10*, 11510-11518.

[9] Y. Yoon, B. Yan, Y. Surendranath, *J. Am. Chem. Soc.* **2018**, *140*, 2397-2400.

[10] a) Y. Deng, A. D. Handoko, Y. Du, S. Xi, B. S. Yeo, *ACS Catal.* **2016**, *6*, 2473-2481; b) S. Jiang, K. Klingan, C. Pasquini, H. Dau, *Journal of Chemical Physics* **2019**, *150*; c) G. Niaura, *Electrochim. Acta* **2000**, *45*, 3507-3519; d) A. Singhal, M. R. Pai, R. Rao, K. T. Pillai, I. Lieberwirth, A. K. Tyagi, *Eur. J. Inorg. Chem.* **2013**, *2013*, 2640-2651.

[11] a) K. G. Schmitt, A. A. Gewirth, *J. Phys. Chem. C* **2014**, *118*, 17567-17576; b) T. Kottakkat, K. Klingan, S. Jiang, Z. P. Jovanov, V. H. Davies, G. A. M. El-Nagar, H. Dau, C. Roth, *ACS Appl. Mater. Interfaces* **2019**, *11*, 14734-14744.
